# Competitive Hybridization of a Microarray Identifies CMKLR1 as an Up-Regulated Gene in Human Bone Marrow-Derived Mesenchymal Stem Cells Compared to Human Embryonic Fibroblasts

**DOI:** 10.3390/cimb44040102

**Published:** 2022-03-28

**Authors:** Hee-Yeon Cho, Sooho Lee, Ji-Hong Park, Yoon Hae Kwak, HaeYong Kweon, Dongchul Kang

**Affiliations:** 1 Ilsong Institute of Life Science, Hallym University, Beodeunaru-ro 55, Seoul 07247, Korea; 429jho@naver.com (H.-Y.C.); navy07@hanmail.net (S.L.); flowjh@hallym.ac.kr (J.-H.P.); 2 Department of Biomedical Gerontology, Hallym University Graduate School, Chuncheon 24252, Korea; 3 Department of Orthopaedic Surgery, Asan Medical Center, Ulsan University College of Medicine, Seoul 05505, Korea; drkwak1215@gmail.com; 4 Industrial Insect and Sericulture Division, National Institute of Agricultural Sciences, RDA, Wanju-gun 55365, Korea; hykweon@korea.kr

**Keywords:** hBMSC, hEF, microarray, CD70, CD339, CMKLR1, RARRES2, chemotaxis

## Abstract

Mesenchymal stem cells (MSCs) have been widely applied to the regeneration of damaged tissue and the modulation of immune response. The purity of MSC preparation and the delivery of MSCs to a target region are critical factors for success in therapeutic application. In order to define the molecular identity of an MSC, the gene expression pattern of a human bone marrow-derived mesenchymal stem cell (hBMSC) was compared with that of a human embryonic fibroblast (hEF) by competitive hybridization of a microarray. A total of 270 and 173 genes were two-fold up- and down-regulated with FDR < 0.05 in the hBMSC compared to the hEF, respectively. The overexpressed genes in the hBMSC over the hEF, including transcription factors, were enriched for biological processes such as axial pattern formation, face morphogenesis and skeletal system development, which could be expected from the differentiation potential of MSCs. CD70 and CD339 were identified as additional CD markers that were up-regulated in the hBMSC over the hEF. The differential expression of CD70 and CD339 might be exploited to distinguish hEF and hBMSC. CMKLR1, a chemokine receptor, was up-regulated in the hBMSC compared to the hEF. RARRES2, a CMKLR1 ligand, stimulated specific migration of the hBMSC, but not of the hEF. RARRES2 manifested as ~two-fold less effective than SDF-1α in the directional migration of the hBMSC. The expression of CMKLR1 was decreased upon the osteoblastic differentiation of the hBMSC. However, the RARRES2-loaded 10% HA-silk scaffold did not recruit endogenous cells to the scaffold in vivo. The RARRES2–CMKLR1 axis could be employed in recruiting systemically delivered or endogenous MSCs to a specific target lesion.

## 1. Introduction

MSCs (mesenchymal stromal cells) in bone marrow provide a niche for hematopoietic stem cells (HSCs) and signals for HSC maintenance and differentiation [1]. The culture of bone marrow aspirate yields fibroblast-like colonies (CFU-F, colony forming unit-fibroblast) that are capable of plastic adherent growth [2]. Bone marrow-derived MSCs (BMSCs) are multipotent cells that can be differentiated into the trilineage of osteoblasts, chondroblasts and adipocytes [3]. The differentiation potential of MSCs is further expanded to muscle cells, endothelial cells and neuronal cells [4]. The trilineage differentiation potential and expandability in in vitro culture have been found in cells that were isolated from various other tissues including adipose tissue, dental pulp, umbilical cord, Wharton jelly and circulation [4]. MSCs residing in diverse tissues are supposed to be involved in the regeneration of mesenchymal-origin tissues and in angiogenesis in vivo [5]. MSCs also retain immunomodulatory functions in transplantation, inflammation and tumor settings [6]. Although stemness that is characterized by self-renewal capacity and multilineage differentiation potential in vivo has not been definitively proven for each of them, these cells have been collectively called mesenchymal stem cells (MSCs) by convention [7]. 

MSCs have been exploited in therapeutic applications for tissue regeneration and modulation of immune response because of their multilineage differentiation potential, immunomodulatory function, simplicity in harvest, expandability in in vitro culture and lack of serious ethical problems [8]. Although various MSC-based therapies have widely been on clinical trial or in practice, the outcome is not as desirable as expected in many cases [8]. The insufficient therapeutic efficacy might be ascribed to the contamination or heterogeneity of MSC preparations as well as inefficient cell delivery to a specific lesion [8,9]. 

MSCs are isolated from diverse tissues, but their differentiation potential to a specific lineage is not equivalent [8,10]. Further, an MSC preparation from a tissue shows heterogeneity unless a single colony is selected for further expansion. The investigation of the molecular identity of MSCs and the search for markers that could be useful in distinguishing MSCs from other cells have been attempted by several groups [11,12]. An analysis of gene expression pattern has found genes that are differentially expressed or epigenetically modified in MSCs from various tissues clustered together compared to non-MSCs, including fibroblasts [13,14]. However, there are significant differences in gene expression pattern among MSCs depending on the differentiation potential to a specific lineage and the tissue sources, which makes it difficult to definitely determine the molecular definition of the cells [15].

The International Society of Cell and Gene Therapy (ISCT^®^) recommends minimal criteria for MSCs. In addition to trilineage differentiation potential, it includes expression of cell surface markers including CD73, CD90 and CD105 and lack of expression of hematopoietic and endothelial markers [7]. Fibroblasts are one of the contaminations often seen in MSC preparations, because the fibroblast shares morphology and plastic adherent growth with the MSC and resides around the MSC location in the bone marrow and other tissues [16]. In addition, surface markers CD73, CD90 and CD105 are also equally expressed in the fibroblast and MSC, which makes an MSC preparation devoid of fibroblasts challenging [17]. Since the differentiation potential of the fibroblast is more restricted than the MSC, fibroblast contamination would significantly interfere with the therapeutic efficacy of an MSC preparation [16]. Thus, searching for new CD markers to distinguish the MSC from the fibroblast has been attempted by various groups. CD106 and CD146 are found to be highly expressed in the MSC, while CD10 and CD26 expression is high in the fibroblast in comparison with the other cells [18]. Recently, CD200 and CD228 have been found to be differentially expressed between the two cells [19]. Once the expression pattern of these new markers is confirmed to be consistent in various MSC preparations, they could be included in an MSC-specific surface marker panel. 

The recruitment of MSCs to a specific lesion is another barrier to their therapeutic application in the regeneration of damaged tissue or the modulation of immune response [9,20]. A fresh MSC preparation or ex vivo expanded cells should be locally or systemically delivered to a specific lesion. Otherwise, endogenous MSCs should be mobilized and migrated to a target organ. However, neither the local nor systemic delivery of MSCs to a specific lesion is an efficient process, which is a significant drawback in the medical application of the cells. The chemokine–chemokine receptor axis in MSCs and the transendothelial/interstitial migration of MSCs have been actively studied in order to facilitate MSC delivery. The CXCL12 (SDF-1)–CXCR4 axis has been reported to stimulate MSC migration to injury sites including bone fractures [21]. MSCs or endogenous cells can also be recruited into CXCL12-loaded 3-D scaffolds in vitro and in vivo [22,23]. In addition, the chemerin (RARRES2 gene product)–CMKLR1 (chemokine-like receptor 1) axis has been reported to activate MSC migration to cancer tissue and damaged liver lesions [24,25]. Additional chemokine receptors that are constitutively expressed or induced by inflammatory cytokines including TNF-α are also reported in MSCs, and their functions in MSC migration are being investigated [9,26]. 

CMKLR1 is a G protein-coupled receptor that binds RARRES2 and resolvin E1 [27]. RARRES2s are processed products of the RARRES2 gene in which the N-terminal signal peptide and multiple positions of the C-terminal amino acids are enzymatically cleaved [28]. Among them, RARRES2 S157 is the most active ligand for its receptors, while RARRES2 A155 antagonizes RARRES2 S157 binding. CMKLR1 functions through Gα i/o and stimulates calcium mobilization, the inhibition of cAMP accumulation, and signaling pathways involving Syk, Erk1/2, p38 MAPK and Akt upon RARRES2 binding [27]. The RARRES2–CMKLR1 axis regulates inflammatory process via activating the chemotaxis of macrophage and natural killer cells, modulates energy metabolism and promotes adipogenesis and angiogenesis depending on the cell context [29,30]. RARRES2 also binds to CCRL2 and GPR1 (RARRES2 receptor 2). Binding to GPR1 activates arrestin recruitment and receptor endocytosis, which suggests a role as a decoy receptor [31]. CMKLR1 also binds resolvin E1, an anti-inflammatory eicosapentaenoic acid derivative. The resolvin E1 interaction with CMKLR1 is known to participate in the resolving stage of inflammation [32]. 

In order to characterize the molecular identity of MSC, the gene expression pattern of the human bone marrow-derived MSC (hBMSC) was compared with that of the human embryonic fibroblast (hEF) by competitive hybridization of a microarray. The STRING functional protein association network and Gene Ontology were analyzed using the differentially expressed genes between the two cells. The chemokine receptor CMKLR1 was found to be overexpressed in the hBMSC compared to the hEF. The specific migration of the hBMSC toward RARRES2 in vitro, the differentiation-associated CMKLR1 expression, and the recruitment of endogenous cells into a RARRES2-loaded 3-D scaffold were examined in this study.

## 2. Materials and Methods

### 2.1. Cell Culture

The hBMSCs (ScienCell Research Laboratories, Carlsbad, CA, USA) were grown in α-MEM (Life Tech., Carlsbad, CA, USA) supplemented with 16.5% fetal bovine serum (FBS, Lonza, Basel, Switzerland), 100 units/mL penicillin and 100 µg/mL streptomycin (HyClone, Logan, UT, USA). Multipotency and expression of MSC-specific CD markers proposed by International Society for Cellular Therapy (ISCT^®^) were previously confirmed for the hBMSC employed in this experiment [19,33]. Human embryonic fibroblasts (hEF) [19,34] were grown in Dulbecco’s modified Eagle’s medium (DMEM) (Life Tech) supplemented with 10% FBS, L-glutamine (Life Tech), 100 units/mL penicillin and 100 μg/mL streptomycin. Both hBMSC and hEF were incubated at 37 °C in a humidified 5% CO_2_ incubator. The hBMSCs and hEFs at ~80% confluency were subcultured by trypsinization (0.25% trypsin-EDTA, Welgene, Seoul, Korea) and replated at 5 × 10^5^ cells on a 10- cm culture dish every 3–4 days.

### 2.2. Microarray Analysis

The gene expression profile of the hBMSC was compared with the hEF by competitive hybridization of Agilent Human Gene Expression 4x44K v2 Microarray (Agilent Technology, Palo Alto, CA, USA) (ebiogen, Seoul, Korea). Total RNA was extracted from 70–80% confluent hBMSCs (passage 3–4) and hEFs (passage 14–16) with the TRIreagent Total RNA isolation solution (GeneAll, Seoul, Korea). Synthesis of target cRNA probes and hybridization were performed using Agilent’s Low RNA Input Linear Amplification kit with total RNA (5 µg each) according to the manufacturer’s instructions (Agilent Technology). The hybridized images were scanned using Agilent’s DNA microarray scanner and quantified with Feature Extraction Software. All data normalization was performed using GeneSpringGX 7.3 (Agilent Technology). The averages of normalized ratios were calculated by dividing the average of normalized signal channel intensity by the average of normalized control channel intensity. Functional annotation of genes was performed according to Gene Ontology^TM^ Consortium (http://www.geneontology.org/index.shtml, accessed on 6 September 2016) by GeneSpringGX 7.3. Gene classification was based on searches using BioCarta (http://www.biocarta.com/, accessed on 6 September 2016), GenMAPP (http://www.genmapp.org/, accessed on 6 September 2016), DAVID (http://david.abcc.ncifcrf.gov/, accessed on 6 September 2016), and Medline databases (http://www.ncbi.nlm.nih.gov/) (accessed on 6 September 2016 for all). Biological processes of genes with 2-fold differences were analyzed in Gene Ontology Panther Classification System (http://www.pantherdb.org/, accessed on 15 January 2022). STRING analysis at https://string-db.org/ was performed to analyze the gene network, clustering and enrichment of gene ontologies and pathways (accessed on 2 September 2021).

### 2.3. Reverse Transcription Polymerase Chain Reaction (RT-PCR)

The TRIreagent Total RNA isolation solution was used to extract total RNA from hBMSC and hEF cells grown to 70–80% confluence. The total RNA (5 µg) was used in a 20 µL reverse transcription reaction with GoScript reverse transcription system (Promega, Madison, WI, USA), dNTPs, and an oligo (dT) primer according to the vendor’s protocol. The RT reaction was diluted by addition of 80 µL DW, and 2 µL of the diluent was used in a PCR reaction. PCR was carried out in a 20 µL reaction with G-Taq polymerase system (Cosmo Genetech, Seoul, Korea) with parameters of one cycle at 95 °C for 3 min followed by 35 cycles of 95 °C for 30 s, 56 °C for 30 s and 72 °C for 1 min, and a cycle at 72 °C for 10 min. Amplicons were resolved by 1.5% agarose gel electrophoresis and visualized by EtBr. RT-PCR band intensity was quantified with ImageJ software. Real-time PCR was carried out with the cDNA diluent (2 µL) using the SYBR^Ⓡ^ Green PCR Kit (Qiagen, Hilden, Germany) in Light Cycler 2.0 (Roche, Basel, Switzerland) as directed by the manufacturer’s protocol. PCR was carried out with the following cycling protocol: 1 cycle of 95 °C for 10 min, followed by 45 cycles of 95 °C for 10 s, 56 °C for 5 s, and 72 °C for 20 s. Signal intensity of target genes was normalized against that of β-Actin. The primer sequences used and expected amplicon sizes are listed in Supplementary Table S1.

### 2.4. Western Blot Analysis

Cells grown to 70–80% confluence were harvested by scraping and lysed in RIPA buffer (50 mM Tris-HCl pH 7.5, 150 mM NaCl, 1% NP-40, 0.5% sodium deoxycholate, 1 mM EDTA, and 0.1% SDS, freshly supplemented with 1 mM DTT and protease inhibitor cocktails). Protein concentration was measured by BCA Protein Assay Reagent (Pierce, Rockford, IL, USA). Protein samples (25 µg) were resolved by 10% SDS-PAGE and electroblotted onto a nitrocellulose membrane (PALL Corporation, Port Washington, NY, USA). Immunodetection on the blot was carried out as previously described [35] and protein bands were visualized with ChemiDoc^TM^ MP System (BioRad, Hercules, CA, USA) after ECL treatment (Advansta, Menlo Park, CA, USA). Primary antibodies used in the immunodetection were anti-CMKLR1 antibody (Novus Biologicals, Centennial, CO, USA) and anti-β-Actin antibody (Santa Cruz Biotech, Santa Cruz, CA, USA), and a secondary antibody was HRP-conjugated anti-rabbit antibody (Invitrogen, Carlsbad, CA, USA). β-Actin was probed to monitor protein loading on the blot. Intensity of detected bands was analyzed with Image Lab software implemented in ChemiDoc^TM^ MP System.

### 2.5. Flow Cytometry

Cell surface markers of hBMSC and hEF were analyzed by labeling with specific fluorescence-conjugated antibodies followed by flow cytometry. Cells were trypsinized, resuspended in PBS containing 2% FBS, and stained with the following antibodies for 30 min at 4 °C: FITC-conjugated anti-CD70 (BioLegend, San Diego, CA, USA), FITC-conjugated anti-CD321 (BioLegend), PE-conjugated anti-CD339 (BD Bioscience, San Jose, CA, USA), FITC-conjugated mouse IgG1 (BioLegend) and PE-conjugated mouse IgG3 (BD Bioscience). Flow cytometry was performed on FACSCalibur^TM^ (BD Biosciences) and analyzed with CellQuest Pro^TM^ software (version 5.2.1, BD Biosciences).

### 2.6. Transwell Migration Assay

Chemotactic migration was examined by transwell migration assay with transwell inserts with 8 µm pores in a 24-well plate (Falcon, Durham, NC, USA). Membrane support of an insert well was coated with 10 µL 0.1% gelatin and dried for 30 min at RT. hBMSCs and hEFs (5 × 10^4^ cells in 200 µL serum- and antibiotics-free α-MEM) were inoculated into the transwell insert and the outer chamber was filled with 500 µL of serum- and antibiotics-free α-MEM with or without chemotactic factors. Chemotactic reagents used were 100 ng/mL SDF-1α (CHM-262, Prospec, East Brunswick, NJ, USA), 100 ng/mL RARRES2 (PRO-788, Prospec), and 100 ng/mL Substance P (Sigma Aldrich, St. Louis, MO, USA). Cells were allowed to migrate by incubation for 8 h in 37 °C/5% CO_2_ incubator. Transwell inserts were fixed by immersion in PBS-buffered 3.7% formaldehyde and stained with 0.23% crystal violet (Sigma Aldrich). Detached membrane support was mounted on a glass slide and examined under a microscope at 200 × magnification (Olympus, Tokyo, Japan). Cells were counted from pictures taken from four randomly chosen fields and the number of cells was averaged for the number of migrated cells/field.

### 2.7. Osteoblastic Differentiation of hBMSC

hBMSC younger than passage eight was plated at 4 × 10^3^ cells/well in a 96-well plate or 4 × 10^5^ cells in a 60-mm dish one day prior to treatment of osteoblast differentiation medium (ODM). ODM consisted of 100 nM dexamethasone, 50 µM ascobate-2-phosphate and 10 mM β-glycerophosphate in 10% FBS-supplemented α-MEM [19]. ODM with or without 100 ng/mL RARRES2 was refreshed every 3–4 days up to 14 days. 

### 2.8. MTT and ALP Assays

The hBMSCs were seeded at 4 × 10^3^ cells/well in a 96-well plate one day prior to treatment of ODM with or without 100 ng/mL RARRES2 for cell viability and alkaline phosphatase assays. Cell viability was measured on day 4 of ODM treatment by MTT assay (3-[4,5-dimethylthiazol-2-yl]-2,5-diphenyl tetrazolium bromide, Sigma Aldrich) as described in Huynh et al. [19]. MTT/well (100 µL 0.5 mg/mL in 10% FBS-supplemented α-MEM) was incubated for 3 h at 37 °C. An equal amount of formazan solubilizer (10% SDS in 0.01 N HCl) was added and further incubated at 37 °C overnight. MTT conversion was measured by the absorbance at 570 nm with the reference absorbance at 650 nm with a Multiskan™ GO Spectrophotometer (Thermo Scientific, Rockland, IL, USA). ALP activity was measured in a 96-well plate on day 4 of ODM treatment [19]. After removing medium, a 150 µL ALP reaction mixture containing 140 µL of alkaline buffer (Sigma Aldrich), 10 µL substrate solution (0.225 M p-nitrophenyl phosphate, Fluka, Buchs, Switzerland) and 1.5 mM MgCl_2_ (Sigma Aldrich) was added and incubated for 30 min at 37 °C. The reaction was stopped by transferring a 20 µL fraction of the reaction to 80 µL 0.2 N NaOH pre-aliquoted in a 96-well plate. Absorbance at 405 nm was measured with the Multiskan™ GO Spectrophotometer. ALP activity was normalized with the viable cell amount measured by the MTT assay. 

### 2.9. Alizarin Red S Staining

Calcium deposition on day 14 of osteogenic differentiation was measured by Alizarin red S staining as described in Dubon et al. [19]. Cells were fixed with PBS-buffered 3.7% formaldehyde for 30 min at RT, rinsed with distilled water, and stained with 2% (*w*/*v*) Alizarin red S (Sigma Aldrich) dissolved in distilled water (pH 4.2, adjusted with 10% NH_4_OH, Sigma Aldrich) for 20 min. Stained cells were washed with distilled water and an image of the mineralization was taken. Then, the dye was eluted with 10% (*w*/*v*) cetylpyridinium chloride monohydrate (Sigma Aldrich) in 10 mM sodium phosphate (pH 7.0; Sigma Aldrich) for 1 h at RT, and the absorbance of the eluent was measured at 570 nm using the Multiskan™ GO microplate reader.

### 2.10. In Vivo Cell Recruitment to Transplanted HA-Silk Scaffold

The chemotactic capacity of RARRES2 in vivo was assessed by transplanting a sponge-type silk 3-D scaffold containing 10% hydroxyapatite (HA) with or without RARRES2 into a rat tibia diaphysis defect. Male Sprague Dawley rats (RaonBio, Yongin, Korea) were maintained in an environment-controlled room with a lighting cycle of 12 h light/12 h dark cycle, temperature of 18–26 °C and humidity in the 30–70% range. A 10% HA-silk scaffold (3-mm diameter) was loaded with RARRES2 by absorbing 5 µL of 100 µg/mL RARRES2 prior to operation. The tibias of both hind limbs of a rat (15 week old, 370–440 gm) anesthetized by inhalation of isoflurane (Hana Pham Co., Ltd., Seoul, Korea) were exposed and a 3 mm-diameter defect was introduced by a drill [36]. An HA-silk scaffold was installed into the tibia defect to fill the gap. Then, the surgery site was closed with a black silk suture (Ailee Co., Ltd., Busan, Korea) and ceftriaxone (4 mg in 100 μL) was intradermally injected for prophylactic purpose. Five rats were used in the experiment. A PBS-soaked scaffold was transplanted into the right limb and a RARRES2-soaked one into the left limb. Radio-opaqueness was monitored weekly by X-ray radiography with EZX-60 potable dental X-ray (Genoray Co., Ltd., Sungnam, Korea). Animal care and operating procedures were approved by the Hallym University Medical Center Institutional Animal Care and Use Committee, Korea (HMC2012-0-1026-1).

### 2.11. Trichrome Staining

Cell migration and bone tissue formation were examined by Trichrome staining of transplanted scaffold with Masson Trichrome Stain Kit (Sigma Aldrich) according to the manufacturer’s procedure. A lower limb with an implanted scaffold was removed by surgical dissection and the adjoined muscle was removed. The cleaned tibia was fixed with PBS-buffered 3.7% formaldehyde at 4 °C overnight and decalcified by incubation twice in Calci-Clear Rapid solution (National Diagnostics, Atlanta, GA, USA) at 4 °C for 8 h each. The fragment of bone with the scaffold was cut with a scalpel and embedded in paraffin. The paraffin-embedded tissue was sectioned into 5 μm-thick slices with a microtome (Leica RM2245, Buffalo Grove, IL, USA) and mounted on glass slides. Deparaffinized slides were stained with standard protocol of the Masson Trichrome Stain Kit and examined under a microscope at 40× and 200× magnification (Olympus).

### 2.12. Statistical Analysis

Quantitative data was analyzed with unpaired two-tailed Student’s *t*-test or one-way ANOVA implemented in Microsoft Excel. Pearson correlation, confidence interval, and probability were calculated with R. Benjamini–Hochberg false discovery rate (FDR) was calculated by *t*-test probability x number of genes in microarray panel/rank in probability. Differences with *p* values of less than 0.05 were considered significant.

## 3. Results

### 3.1. Differential Gene Expression between hBMSC and hEF in a Microarray Analysis

**Figure 1:**
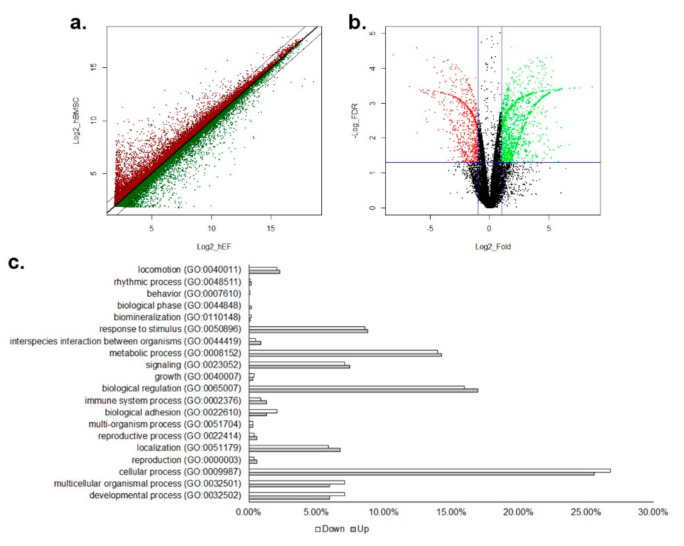
Graphical summary of the microarray results. (**a**) A scatter plot of all data points in the microarray analysis. Red dots represent Log_2_(hBMSC) > Log_2_(hEF) and green dots are the others. Thick solid midline is for Log_2_(hBMSC) = Log_2_(hEF). Two thin solid lines represent genes of two-fold difference. (**b**) A volcano plot for all data points in the microarray analysis. Red dots are genes of two-fold lower expression and FDR < 0.05 and green dots represent two-fold higher expression and FDR < 0.05 in hBMSC. (**c**) Analysis of biological processes of genes with two-fold difference and FDR < 0.05 in Gene Ontology Panther Classification System. Blank bars for two-fold lower expression and solid bars for two-fold higher expression in hBMSC.

**Figure 2:**
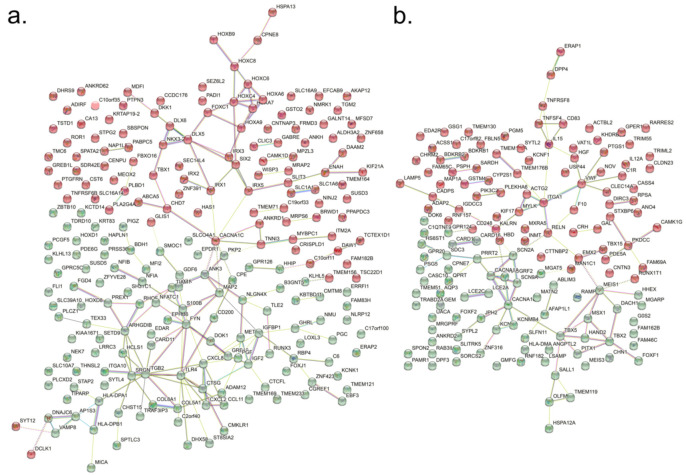
Network analysis and clustering of up- and down-regulated genes with String (https://string-db.org/, accessed on 23 February 2022). Network analysis with two-cluster k-means clustering (red and green) of genes with FDR < 0.05 and two-fold higher expression (**a**) or FDR < 0.05 and two-fold lower expression (**b**) in hBMSC identified by the microarray analysis.

The gene expression pattern of the hBMSC was compared with the hEF by Agilent human 44K microarray analysis in triplication. With a cut-off of *p* < 0.05 and a minimum signal cut-off value of 16, the expression of 1232 and 805 genes was found to be two-fold higher or lower in hBMSC than hEF, respectively (Figure 1a). The application of the cut-off of *p* < 0.05 of FDR found 270 and 173 genes to be two-fold up- and down-regulated in the hBMSC, respectively (Figure 1b, Supplementary Table S2). The proportions of up- and down-regulated genes involved in broad categories of biological processes were not significantly different in the Panther GO Slim biological process analysis (Pearson correlation coefficient = 0.996, 95% confidence interval 0.990–0.999, *p* = 1.284 × 10^−20^) (Figure 1c). The network analysis with STRING and k-means clustering of up-regulated genes showed two major clusters (Figure 2a, Supplementary Table S3). Cluster 1 was highly enriched with genes for face morphogenesis (101.39 folds), pattern formation (101.36 folds) and embryonic skeletal system morphogenesis (101.25 folds). Cluster 2 was enriched with genes associated with the negative regulation of IL-6 production (101.29 folds), neutrophil chemotaxis (101.06 folds) and negative regulation of protein secretion (101.02 folds). Down-regulated genes were also grouped in two clusters: one enriched with genes in KEGG complement/coagulation cascades (101.19 folds), and the other with genes in endocardial cushion development (101.41 folds), embryonic limb morphogenesis (101.19 folds) and action potential (101.16 folds) (Figure 2b, Supplementary Table S4). 

**Figure 3:**
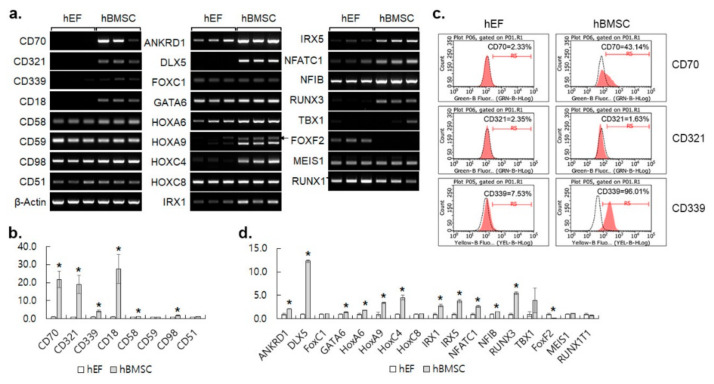
Expression of selected transcription factors and cell-surface markers. (**a**) RT-PCR of 17 transcription factors and 8 CD genes that showed differential expression in the microarray analysis. Results shown are triplicates with three independently isolated samples. (**b**) Quantification of the RT-PCR results for CD genes. Results shown are means ± SDs of fold increase in hBMSC against hEF of the triplicates. * represents *p* < 0.05 in Student’s *t*-test. (**c**) Representative histograms of triplicated flow cytometric analysis of three indicated CDs. Dotted blank lines for signals of negative isotype controls. (**d**) Quantification of the RT-PCR results for selected transcription factors. Results were analyzed and are shown as in (**b**).

Certain cell surface antigens such as clusters of differentiation (CD) genes have been known as markers that characterize MSCs [18]. The expression of CD markers in MSCs including CD90, CD73, CD105, CD200, CD106, and CD228 was verified by RT-PCR (data not shown) and previously by flow cytometry [19]. Additional eight CDs that displayed differential expression in the microarray analysis were analyzed by RT-PCR, and six of them (CD70, 321, 339, 18, 58 and 98) showed significantly different expression between the hBMSC and the hEF (Figure 3a,b). Among them, the cell surface expression of two CDs (CD70 and CD339) was confirmed to be higher in the hBMSC than the hEF by flow cytometry (Figure 3c).

Fifty transcription factors showed a two-fold difference with FDR < 0.05. A total of 35 and 15 of them were high and low in the hBMSC compared to the hEF, respectively. The expression levels of 17 genes were analyzed and 12 of them were found to be significantly different by RT-PCR (Figure 3a,d). The correlation between the microarray and RT-PCR results was 0.88 (95% confidence interval 0.69–0.96, *p* = 3.73 × 10^−6^). The transcription factors were assorted into two clusters (Supplementary Table S5). The enriched biological process in Cluster 1 included pattern specification (101.6 folds), embryonic skeletal system development, and morphogenesis (101.72 folds). The other cluster consisted of the morphogenesis of various organs, the development and differentiation of the mesoderm and mesenchyme (101.38 folds), stem cell differentiation (101.37 folds), skeletal system morphogenesis (101.33 folds) and cartilage development (101.33 folds). The enriched biological processes of differentially expressed transcription factors appeared to reflect the overall difference in gene expression between the hBMSC and hEF. 

### 3.2. CMKLR1 Is Up-Regulated in hBMSC

**Figure 4:**
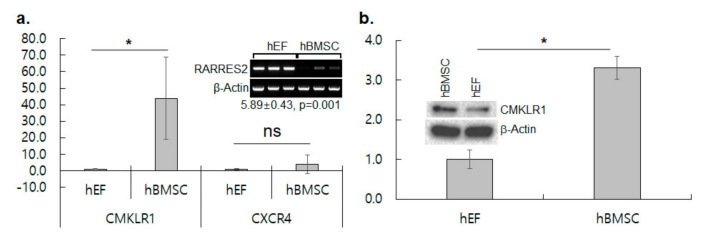
Differential expression of CMKLR1 between hBMSC and hEF. (**a**) Comparison of transcript level by quantitative real-time RT-PCR for CMKLR1 and CXCR4 between hBMSC and hEF. Results shown are means ± SDs of fold increase in hBMSC against hEF of three independent samples. * represents *p* < 0.05 in Student’s *t*-test. Inset: RT-PCR and quantitative analysis of RARRES2 expression shown by fold increase in hEF against hBMSC. RT-PCR shown here was executed with the same cDNA samples as in Figure 3a. (**b**) Comparison of CMKLR1 protein level by western blotting. Results shown are means ± SDs of fold increase in hBMSC against hEF of three independent samples. * represents *p* < 0.05 in Student’s *t*-test. Inset: A representative western blot result for CMKLR1. β-Actin was employed as a loading control.

Chemokine–chemokine receptor associations are critical for MSC migration and proliferation [9]. Among the 40 chemokine receptors, the differential expression of CMKLR1 was detected to be ~36-fold higher in the hBMSC than the hEF in the microarray analysis. The transcript level of CMKLR1 was 43.9 folds (*p* = 0.034), while that of CXCR4 was 4.0 folds (*p* = 0.398) higher in hBMSC in real-time RT-PCR (Figure 4a). The protein level was 3.3 folds higher in the hBMSC than the hEF in western blotting, confirming the differential expression showed in microarray analysis and RT-PCR (Figure 4b). Interestingly, the expression of RARRES2, a ligand of CMKLR1, was detected 68.2 folds (*p* = 2.586 × 10^−5^) higher in the hEF in the microarray analysis and 5.9 folds (*p* = 0.001) higher in semiquantitative RT-PCR (Figure 4a, *Inset*). CMKLR1 was definitively highly expressed in the hBMSC compared to the hEF, while the complementary ligand RARRES2 was highly expressed in the hEF. 

### 3.3. RARRES2 Activates Migration of hBMSC, but Not of hEF

**Figure 5:**
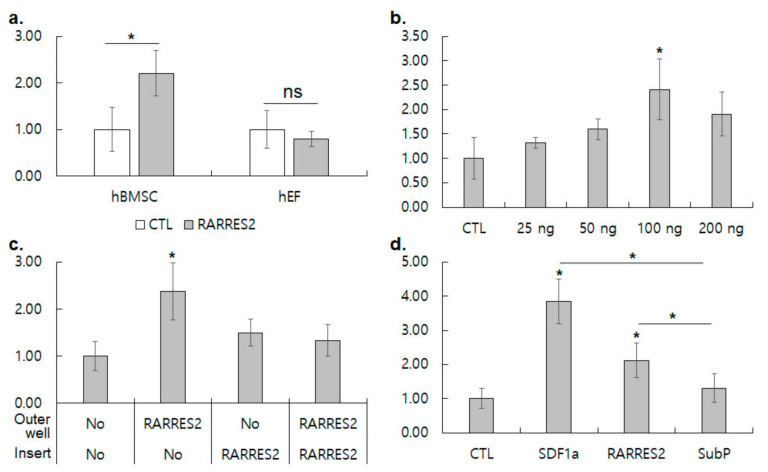
Transwell cell-migration assays for migration of hBMSC by RARRES2, a ligand for CMKLR1. (**a**) Comparison of chemotactic migration of hBMSC and hEF by RARRES2. Results shown are means ± SDs of fold increase in migrated cells/field by 100 ng/mL RARRES2 treatment (filled bars) against untreated control (blank bars) for hBMSC and hEF, respectively. Experiments were repeated five times. (**b**) Concentration-dependent migration of hBMSC was measured by adding indicated amount of RARRES2 in outer wells. Results shown are means ± SDs of fold increase in migrated hBMSCs/field against untreated control in triplicated experiments. (**c**) Directionality of migration was measured by adding 100 ng/mL in the indicated compartments. Results shown are means ± SDs of fold increase in migrated hBMSCs/field against untreated control in quadruplicated experiments. (**d**) Comparison of chemotactic activity of various chemokines was performed by adding indicated chemokines in the outer well. Results shown are means ± SDs of fold increase in migrated hBMSCs/field against untreated control in triplicated experiments. * represents *p* < 0.05 in Student’s *t*-test against untreated control or between two aligned groups.

In order to test the hBMSC-specific chemotactic activity of RARRES2, the chemotactic migration of hBMSCs and hEFs was examined by a transwell cell migration assay. RARRES2 in the outer chamber of a transwell plate (100 ng/mL) increased hBMSC migration by 2.21 ± 0.49 folds (*p* = 0.001), while it did not significantly change the migration of hEFs (0.79 ± 0.16, *p* = 0.307, Figure 5a). Since the specific activity of RARRES2 for hBMSC was verified, we determined the dependence of hBMSC migration on the concentration of RARRES2. The migration of hBMSCs gradually increased with the concentration increase up to 100 ng/mL, but slightly decreased at 200 ng/mL (Figure 5b). Cell migration should be directional toward a specific chemokine. Therefore, we examined the directionality of hBMSC migration by the addition of RARRES2 in the inner and/or outer chamber in a transwell migration assay. The addition of RARRES2 in the outer chamber only increased hBMSC migration by 2.37 ± 0.60 folds, while RARRES2 in the insert chamber only or in both chambers did not significantly increase hBMSC migration (Figure 5c). hBMSC migration to RARRES2 in the outer chamber was significantly higher than RARRES2/RARRES2 and RARRES2/CTL in the respective insert/outer chambers (1.78 and 1.58 folds, *p* = 0.024 and *p* = 0.040, respectively). These results demonstrated that the RARRES2 effect on hBMSC migration was directional. Next, the chemotactic migration of hBMSC by RARRES2 was compared with other chemokines including SDF-1α and substance P (Figure 5d). Although the differential expression of CXCR4 (a receptor for SDF-1α) appeared less prominent than that of CMKLR1 in the microarray and RT-PCR analysis (Figure 4a), SDF-1α increased the chemotactic migration of hBMSCs more strongly than RARRES2 did (3.84 ± 0.65 folds over control, *p* = 0.002). RARRES2 manifested the second highest chemotactic activity for hBMSCs (2.12 ± 0.51 folds, *p* = 0.030). Substance P did not significantly alter the migration of hBMSCs. CMKLR1 did involve in the specific chemotactic migration of hBMSCs, but its activity was not as strong as CXCR4. 

### 3.4. CMKLR1 Expression during Osteoblastic Differentiation of hBMSC

**Figure 6:**
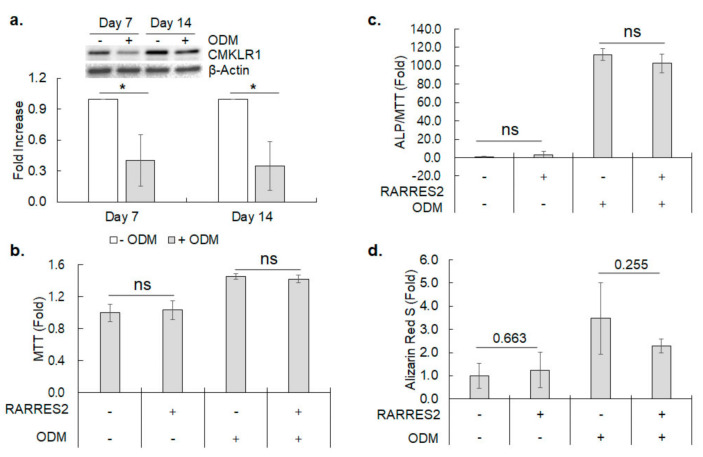
Expression of CMKLR1 on osteogenic differentiation and the effect of RARRES2 on osteogenic differentiation of hBMSC. (**a**) Expression of CMKLR1 protein upon osteogenic differentiation of hBMSC was examined by western blotting. Results shown are means ± SDs of fold increase in ODM-treated hBMSCs (filled bars) against untreated control (blank bars) of three independently prepared samples. Inset: A representative western blot result for CMKLR1 in untreated or ODM-treated cells for indicated days. β-Actin was employed as a loading control. (**b**) Cell viability of differentiating hBMSCs was measured by MTT assay on day four of indicated treatment. Results shown are means ± SDs of fold increase in viability against untreated control in quadruplicated experiments. (**c**) Alkaline phosphatase activity normalized against the cell viability measured by MTT assay. Results shown are means ± SDs of fold increase in alkaline phosphatase activity against untreated control in quadruplicated experiments. (**d**) Alizarin red S staining was performed on day 14 of osteogenic differentiation of hBMSC by indicated treatment. Results shown are means ± SDs of fold increase in Alizarin red S staining against untreated control in triplicated experiments. * represents *p* < 0.05 in Student’s *t*-test against untreated control or between two aligned groups.

hBMSCs can be differentiated into osteoblasts upon treatment of osteogenic medium [3]. Thus, we examined changes in CMKLR1 expression on differentiating osteoblasts. The CMKLR1 protein level was decreased by ~2.5 folds at day 7 and maintained until day 14 after the osteogenic medium treatment (*p* = 0.014 and 0.009, respectively) (Figure 6a). Next, the effect of CMKLR1 activation on the osteogenic differentiation of the hBMSC was examined by inducing osteogenic differentiation in the absence or presence of RARRES2. RARRES2 treatment did not change the cell viability and differentiation parameters of the hBMSC, including alkaline phosphatase activity and calcium deposition (Figure 6b–d). Although CMKLR1 expression obviously decreased upon osteogenic differentiation, RARRES2 did not significantly influence osteogenic differentiation capacity. 

### 3.5. The Effect of RARRES2 on Cell Migration into a 3-D Silk Scaffold In Vivo

**Figure 7:**
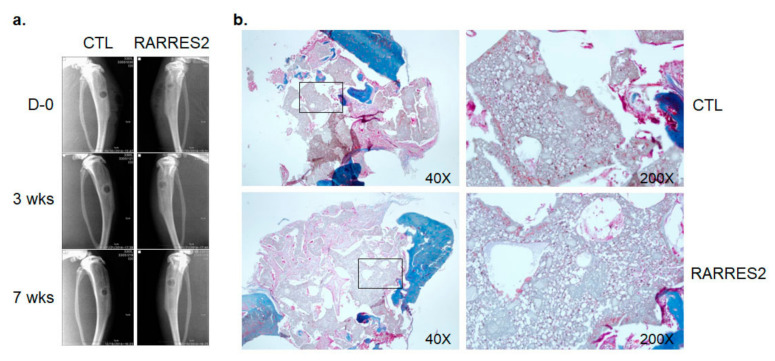
RARRES2-induced cell migration in vivo. In vivo cell migration by RARRES2 was assessed by transplanting 10% HA-silk scaffold into circular tibial damage. (**a**) Representative X-ray radiograms on indicated weeks post-surgery. (**b**) Cell infiltration into the scaffold was examined by microscopic observation after Trichrome staining. Boxed areas in lower magnification (40×) are enlarged to 200× (right panels).

The chemotactic attraction of MSCs to bone injury sites is supposed to promote bone regeneration [21]. Therefore, we examined the effect of RARRES2 on cell migration into an implanted 3-D 10% HA-silk scaffold. The HA-silk scaffold, presoaked with PBS alone or with RARRES2-PBS, was transplanted into a 3 mm-diameter tibia injury in a rat. Bone formation in the scaffold was monitored weekly for seven weeks by X-ray imaging and histology was observed by trichrome staining of recovered scaffold after seven weeks. The radio-opaqueness seemed high in the RARRES2-HA-silk during the weekly post-operative follow-up, but did not significantly increase with time, which suggests that the observed radio-opaqueness should be considered a nonspecific phenomenon (Figure 7a). Moreover, the cell density in the scaffold did not appear different between the two groups in the histological staining (Figure 7b). Although RARRES2 showed chemotactic activity for the hBMSC in vitro, its effect on the migration of bone-forming cells in vivo was not obvious. 

## 4. Discussion

The gene expression pattern between the hBMSC and hEF was compared by competitive hybridization of microarray in order to determine the molecular identity of the hBMSC. The up-regulated genes in the hBMSC compared to the hEF were enriched in the biological processes including axial pattern formation, face development and skeletal system development, which could be anticipated from the differentiation potential of MSCs [37]. Enriched biological processes in the overall gene expression pattern were also observed in the enriched processes of differentially expressed transcription factors. In addition, transcription factors were enriched for biological processes related to stem cell properties. Among the up-regulated transcription factors, NFIB, which is commonly highly expressed in MSCs from bone marrow, adipose tissue and umbilical cord compared to fibroblasts, was also found to be highly expressed in the hBMSCs in this experiment [38]. NFIB stimulates chondrocyte proliferation and the deposition of the extracellular matrix [39]. However, a mouse with an NFIB deficiency develops neurological defects and lung hypoplasia [40], which obscures the role of NFIB in MSC maintenance and lineage-specific differentiation. DLX5, whose expression has been closely correlated with osteogenic differentiation potential among MSCs of different origin [15], was also highly expressed in the hBMSC compared to the hEF. These results suggest that one of the major differences between the hBMSC and hEF involves the regulation of developmental process and differentiation potential, especially osteogenic differentiation. MSCs from bone marrow retain stronger osteogenic and chondrogenic differentiation potential than those from adipose tissue, which might explain the lack of significant gene enrichment associated with adipogenesis and/or adipocyte function [41]. 

The hBMSC expressed MSC-specific CD markers such as CD73, CD90, CD105, but their expression was almost indistinguishable from the hEF [19]. In contrast, CDs including CD228, CD106 and CD200 were found to be highly expressed in the hBMSC over the hEF. Here, two more CDs, CD70 and CD339, were determined to be up-regulated in the hBMSC in comparison with the hEF. CD18 could also be differentially expressed between the two cells. CD70, a ligand of CD27, is a tumor necrosis factor family protein and is involved in T cell development into cytotoxic T cells [42]. CD339, a Notch ligand Jag1, is known to be highly expressed in MSCs and involved in regulation of MSC differentiation into various lineages as well as hematopoiesis [43,44]. Thus, in addition to CD228, CD106 and CD200, both CD70 and CD339 could be novel markers for distinguishing BMSCs from fibroblasts. However, the universal up-regulation of these CD markers in MSCs from various sources and association with stem cell maintenance and differentiation potential remain to be firmly verified.

A chemokine receptor CMKLR1 was found to be overexpressed in the hBMSC compared to the hEF by competitive hybridization of microarray, RT-PCR and western blotting. The expression of CMKLR1 has been reported in bone marrow-derived MSCs and decreases upon treatment with lithium, an osteogenic differentiation enhancer [45]. CMKLR1 is also up-regulated in dermal MSCs from psoriasis patients [46]. CMKLR1 is known to express in immature dendritic cells, macrophages and natural killer cells, and RARRES2, a specific ligand for CMKLR1, stimulates chemotactic migration of the cells [47]. CMKLR1 is also involved in chemotaxis as well as the adipogenic differentiation of MSCs [24,25,30,48]. The RARRES2–CMKLR1 axis has been described to induce the migration of MSCs to esophageal cancer [24]. Recently, the activation of MSC migration by RARRES2 was reported in vitro at the 2-D and 3-D level, and in vivo in a RARRES2-producing liver [25]. The specific migration of the hBMSC, but not the hEF, toward RARRES2 was demonstrated here, which further verifies the role of RARRES2 and CMKLR1 in MSC migration. 

The CXCL12–CXCR4 axis has been known to activate MSC migration into various tissue lesions and transplanted scaffolds [49]. The expression of CXCR4 was not significantly different between the hBMSC and hEF in the microarray analysis (0.96-fold increase in hBMSC over hEF, *p* = 0.830) and quantitative RT-PCR. However, in comparison of the migration potential, the migrated cells by SDF-1α were ~two-fold higher than those by RARRES2. Thus, both SDF-1α and RARRES2 appear to be active chemokines that induce the chemotactic migration of MSCs. RARRES2 is produced by tissue fibroblasts, mast cells and adipocytes [28]. The overexpression of RARRES2 in the hEF compared to the hBMSC was also found by competitive microarray hybridization and verified by RT-PCR. Although SDF-1α is a more effective chemokine than RARRES2 in terms of cell migration, RARRES2 could be more selective for the delivery of MSCs to a specific local lesion. 

In addition to chemotactic activity, the RARRES2–CMKLR1 axis regulates adipogenic differentiation, angiogenesis and myogenesis [27]. RARRES2 increases the adipocytic differentiation of primary BMSC and 3T3-L1 mouse fibroblast [30,48]. The knockdown of RARRES2 or CMKLR1 inhibits adipocytic differentiation but increases osteoblastic differentiation markers [48]. In addition, lithium treatment that enhances the osteoblastic differentiation of MSC decreases CMKLR1 expression [45]. CMKLR1 expression was also decreased in days 7 and 14 of the osteogenic differentiation of the hBMSC. However, RARRES2 treatment showed a weak tendency to decrease osteoblastic differentiation, but not significantly. CMKLR1 expression is decreased by osteoblastic differentiation, but its regulatory role in osteogenic differentiation appears not to be significant.

The chemotactic activity of CXCL12 for MSCs has been widely exploited to recruit MSCs to a specific lesion [49]. The migration of cultured MSCs or endogenous MSCs into SDF-1α-loaded hydrogels or scaffolds was demonstrated in vitro and in vivo [22,23]. A RARRES2-loaded 10% HA-silk scaffold that was implanted into a rat tibial defect failed to recruit endogenous cells into the scaffold. Although a RARRES2-loaded scaffold is not used to recruit inoculated MSCs or endogenous cells, the induced expression of RARRES2 has been shown to attract MSCs in vivo, which demonstrates the MSC chemotaxis toward a sustained RARRES2 source [24,25]. Failure to recruit cells to the scaffold might be ascribed to the transient release of RARRES2 or to the lack of a sufficiently effective concentration of RARRES2, which could be compensated by preincubation with a higher concentration of RARRES2 or a prolonged release of the chemokine. Detailed studies considering these factors should be necessary to assess the MSC-recruiting capacity of RARRES2 in vivo. 

The comparison of the gene expression pattern between the hBMSC and hEF revealed that genes involved in axial patterning, face morphogenesis and skeletal system development were up-regulated in the hBMSC. In addition, CD70 and CD339 were highly expressed in the hBMSC and could be employed to distinguishing hBMSCs from hEFs. The overexpression of the chemokine receptor CMKLR1 and the migration of hBMSCs toward RARRES2 were verified in vitro, which suggests the utility of the RARRES2–CMKLR1 axis in the recruitment of systemically administered MSCs or endogenous cells to a specific lesion. 

## Data Availability

Not applicable.

## Supplementary Materials

The following supporting information can be downloaded at: https://www.mdpi.com/article/10.3390/cimb44040102/s1, Table S1: Sequences of PCR primers; Table S2: List of genes up- and down-regulated more than two folds in hBMSC against hEF and FDR < 0.05. Table S3: List of enriched biological processes with genes up-regulated more than two folds in hBMSC against hEF and FDR < 0.05; Table S4: List of enriched biological processes with genes down-regulated more than two folds in hBMSC against hEF and FDR < 0.05; Table S5: List of enriched biological processes with transcription factors differentially expressed more than 2 folds between hBMSC and hEF and FDR < 0.05.
