# Supplementary material for: Competitive Hybridization of a Microarray Identifies CMKLR1 as an Up-Regulated Gene in Human Bone Marrow-Derived Mesenchymal Stem Cells Compared to Human Embryonic Fibroblasts
Source: Curr Issues Mol Biol. 2022 Mar 28;44(4):1497–512. doi: 10.3390/cimb44040102 (PMC9164045; doi:10.3390/cimb44040102)
Supplement: Supplementary file 1 [file cimb-44-00102-s001.zip › cimb-1631853-supplementary.pdf]

Supplementary Table S1. Sequences of PCR primers.

Gene	GenBank Accession	Forward	Reverse	Product Length (bp)
CD70	NM_001252.4	GTCACTTGGGTGGGACGTAG	AGTGAGGTTGGTGCAGAGTG	364
CD321	NM_001113207.1	CTCGCTTCCTGAACTCCGTT	TAGTTGCGAGCCCCAGTGTA	288
CD339	NM_000214.2	CTGTCCATGCAGAACGTGAAC	CAGCCTTGTCGGCAAATAGC	596
CD18	NM_000211.4	GATGACGGCTTCCATTTCGC	TGGGGATGATCTCGGTGAGT	226
CD58	NM_001779.2	TATCCCAAGCAGCGGTCATT	ATTGGAGTTGGTTCTGTCTGGT	136
CD59	NM_203330.2	ACAACCCGCTTGAGGGAAAA	TGCTGCCAGAAATGGAGTCA	141
CD98	NM_003486.6	CGTCAATGGGTCCCTGTTCA	CTTCTGACACAGGACGGTCG	481
CD51	NM_002210.4	GGGACTCCTGCTACCTCTGT	GCTCCAAACCACTGATGGGA	315
ANKRD1	NM_014391.2	AACCTGTGGATGTGCCTACG	AGTCTCACCGCATCATGCAA	436
DLX5	NM_005221.5	TTCCAAGCTCCGTTCCAGAC	GTAATGCGGCCAGCTGAAAG	409
FOXC1	NM_001453.2	TCGGCTTGAACAACTCTCCAG	ACAGTCGTAGACGAAAGCTCC	104
GATA6	NM_005257.5	TGGATTGTCCTGTGCCAACT	CCTGAGGCTGTAGGTTGTGT	300
HOXA6	NM_024014.3	AGTCTCCCGGACAAGACGTA	GGCTGCGTGGAATTGATGAG	530
HOXA9	NM_152739.3	TGGGCAACTACTACGTGGAC	GTTGGCTGCTGGGTTATTGG	578
HOXC4	NM_014620.5	CCCGAGAAATCACAGTCGCT	ACTTTGGTGTTGGGGAGTCG	395
HOXC8	NM_022658.3	CGCACCACGTTCAAGACTTC	TCCAAGGTCTGATACCGGCT	331
IRX1	NM_024337.3	TAGCCACGGACTGTACACCT	AGGTCTCTCTCTGGGAGCGT	246
IRX5	NM_005853.5	CCTATCCGCAGGGCTACTTG	CTCAGCTCCTCCTGCTTCG	662
NFATC1	NM_172387.2	TTCGAGTTTAACCAGCGCGA	GCCCAAGCACGAGGTTATCT	300
NFIB	NM_005596.3	GACAAAGTCTGGCGTCTGGA	GGAGGTGGAGTTCGAGTTGA	713
RUNX3	NM_001031680.2	CTACCACCGAGCCATCAAGG	GGGTCGGAGAATGGGTTCAG	240
TBX1	NM_080647.1	GGTCACTGCCTACCAGAACC	GGGCAATAGTCGTAGGAGCC	666
FOXF2	NM_001452.1	GCCGCCCTACTCGTACATC	ACCACGCGGTGGTACATGG	333
MEIS1	NM_002398.2	CACGGGACTCACCATCCTTC	CACTCATAGGTCCTGGTGCTC	194
RUNX1T1	NM_004349.3	TGCAGCAGTTTGGCAATGAC	TGGTTCTGTCTGGAGTTCGC	348
CMKLR1	NM_001142343	AGGGAAGAGCAAGAACACGA	CTTTCATCCAAACCAGACAC	176
RARRES2	NM_002889.3	GGAATTTCACAAGCACCCGC	AAGGCGAACTGTCCAGGGAA	363
β-Actin	NM_001101.5	GGCATCGTGATGGACTCCG	GCTGGAAGGTGGACAGCGA	612

Supplementary Table S2. List of genes up- and down-regulated more than two folds in hBMSC against hEF and FDR<0.05.

Systematic	Genbank	Gene Symbol	hBMSC/ hEF (Norm)	Log2 (norm)	P-value	FDR
A_23_P170649	NM_153225	C8orf84	402.807	8.654	0.000	0.047
A_23_P171074	NM_004867	ITM2A	186.550	7.543	0.000	0.047
A_32_P66881	NM_138554	TLR4	148.599	7.215	0.000	0.047
A_33_P3235147	NM_005221	DLX5	147.837	7.208	0.000	0.047
A_33_P3368453	NM_000247	MICA	108.207	6.758	0.000	0.047
A_23_P386320	NM_033316	MFI2	71.460	6.159	0.000	0.048
A_23_P152235	NM_024336	IRX3	64.779	6.017	0.000	0.048
A_23_P9135	NM_033655	CNTNAP3	64.505	6.011	0.000	0.047
A_24_P766716	NM_001142343	CMKLR1	60.880	5.928	0.000	0.048
A_33_P3414037	NR_120661.1	TSPAN14-AS1	60.316	5.914	0.000	0.046
A_33_P3363420	NM_174938	FRMD3	57.235	5.839	0.000	0.047
A_23_P348636	NM_001454	FOXJ1	56.110	5.810	0.000	0.046
A_33_P3240843	NM_144649	TMEM71	52.640	5.718	0.000	0.046
A_33_P3242883	NM_005222	DLX6	51.514	5.687	0.000	0.046
A_23_P121480	NM_001004196	CD200	48.324	5.595	0.000	0.047
A_23_P81676	NM_198239	WISP3	45.669	5.513	0.000	0.046
A_32_P140489	NM_001001557	GDF6	43.378	5.439	0.000	0.047
A_23_P161218	NM_014391	ANKRD1	42.128	5.397	0.000	0.044
A_23_P383986	NM_015892	CHST15	41.543	5.377	0.000	0.027
A_23_P86653	NM_002727	SRGN	40.781	5.350	0.000	0.047
A_23_P329573	NM_000211	ITGB2	38.339	5.261	0.000	0.034
A_23_P27013	NM_024017	HOXB9	38.259	5.258	0.000	0.017
A_23_P167129	NM_022475	HHIP	34.610	5.113	0.000	0.046
A_33_P3321382			33.819	5.080	0.000	0.046
A_33_P3249394	NM_001129827	CACNA1C	33.335	5.059	0.001	0.045
A_23_P92928	NM_000065	C6	32.503	5.022	0.001	0.045
A_23_P140384	NM_001911	CTSG	31.838	4.993	0.001	0.045
A_23_P423462	NR_027245	C18orf20	31.570	4.980	0.001	0.045
A_24_P928969	NM_001145369	PTPN3	31.422	4.974	0.001	0.046
A_33_P3385161	XM_001715900	EFCAB9	31.386	4.972	0.000	0.043
A_23_P69537	NM_006681	NMU	31.041	4.956	0.000	0.042
A_23_P323761	NM_025228	TRAF3IP3	30.251	4.919	0.001	0.045
A_23_P353149	NM_178552	C22orf33	29.228	4.869	0.001	0.045
A_33_P3363425	XR_017002	FRMD3	29.208	4.868	0.000	0.046
A_23_P125717	NM_004538	NAP1L3	29.190	4.867	0.000	0.018
A_33_P3220090	NM_001076781	ZNF391	28.990	4.857	0.001	0.045
A_33_P3300975	NM_014620	HOXC4	28.695	4.843	0.000	0.046
A_23_P217901	NM_001113207	TSTD1	28.688	4.842	0.001	0.045
A_24_P52697	NR_002196	H19	28.378	4.827	0.001	0.045
A_24_P280983	NR_002795	HOXA11AS	28.047	4.810	0.000	0.029
A_33_P3332937	XM_001716126	LOC650794	27.434	4.778	0.000	0.047
A_23_P42868	NM_000596	IGFBP1	27.378	4.775	0.001	0.045
A_23_P4714	NM_006533	MIA	26.919	4.751	0.000	0.025

A_23_P92334	NM_024943	TMEM156	25.624	4.679	0.001	0.045
A_24_P106624	NM_005924	MEOX2	25.619	4.679	0.001	0.046
A_33_P3291871			25.557	4.676	0.001	0.045
A_33_P3215968			24.794	4.632	0.001	0.046
A_23_P66635	NM_002986	CCL11	24.710	4.627	0.001	0.046
A_23_P7965	NM_002630	PGC	24.673	4.625	0.000	0.036
A_33_P3213082	AK024093	LOC101927770	24.275	4.601	0.000	0.023
A_23_P403898	NM_002829	PTPN3	24.159	4.594	0.000	0.024
A_23_P94319	NM_014867	KBTBD11	23.908	4.579	0.000	0.046
A_33_P3307197	NM_020440	PTGFRN	23.432	4.550	0.000	0.025
A_23_P423108	NM_182499	TDRD10	23.155	4.533	0.000	0.021
A_33_P3255587	AK056732	LOC144817	23.142	4.532	0.001	0.046
A_23_P5903	NM_016354	SLCO4A1	23.124	4.531	0.001	0.047
A_33_P3441021	NM_001136534	TMEM233	22.959	4.521	0.000	0.032
A_23_P300090	NM_032128	SLC10A7	22.378	4.484	0.001	0.048
A_33_P3246418	NM_005586	MDFI	22.119	4.467	0.000	0.045
A_24_P658427	NM_005596	NFIB	21.830	4.448	0.000	0.045
A_23_P128362	NM_206819	MYBPC1	21.634	4.435	0.001	0.047
A_33_P3345225			21.608	4.434	0.001	0.047
A_33_P3260654	BC028083	TRBV5-4	21.526	4.428	0.000	0.046
A_24_P220822	NM_174952	C4orf37	21.104	4.399	0.001	0.046
A_33_P3394699	AK127499	LOC100129498	20.503	4.358	0.000	0.042
A_24_P179467	NM_005071	SLC1A6	20.274	4.342	0.001	0.048
A_33_P3221748	NM_001031680	RUNX3	20.207	4.337	0.000	0.046
A_33_P3289121	NM_032411	C2orf40	20.177	4.335	0.000	0.039
A_23_P143526	NM_006272	S100B	20.148	4.333	0.000	0.023
A_33_P3215298	XM_001715728	ANKRD62	19.825	4.309	0.001	0.048
A_33_P3613000	NM_001105539	ZBTB10	19.738	4.303	0.000	0.048
A_33_P3271325	NR_002790	PER4	19.437	4.281	0.000	0.046
A_33_P3216714	NM_014787	DNAJC6	19.435	4.281	0.000	0.046
A_33_P3249982	XR_001752012.1	ABCC11	19.262	4.268	0.001	0.048
A_23_P7727	NM_001884	HAPLN1	18.756	4.229	0.000	0.020
A_24_P822931	BC034811	TRPC5OS	18.696	4.225	0.001	0.048
A_33_P3406171	NM_080832	PABPC5	18.478	4.208	0.000	0.040
A_23_P159974	NM_033495	KLHL13	18.428	4.204	0.000	0.035
A_23_P38167	NM_022036	GPRC5C	18.264	4.191	0.000	0.046
A_33_P3405728	NM_004572	PKP2	18.052	4.174	0.001	0.049
A_23_P386254	NM_001189	NKX3-2	18.007	4.171	0.001	0.048
A_32_P160045	NM_152665	TCTEX1D1	17.605	4.138	0.001	0.049
A_23_P168847	NM_172366	FBXO16	17.459	4.126	0.000	0.023
A_24_P929388	NM_138390	TMEM169	17.241	4.108	0.000	0.028
A_23_P357207	NM_138409	MRAP2	17.226	4.106	0.001	0.049
A_33_P3295203	NM_001523	HAS1	15.893	3.990	0.000	0.018
A_23_P150609	NM_000612	IGF2	15.868	3.988	0.000	0.021
A_33_P3214665	NM_002374	MAP2	15.715	3.974	0.001	0.045
A_33_P3224780	XM_011510659.2	TMEM37	15.600	3.963	0.000	0.048
A_23_P500998	NM_152739	HOXA9	15.449	3.949	0.000	0.046
A_23_P258769	NM_002121	HLA-DPB1	15.398	3.945	0.000	0.046

A_23_P421032	NM_174977	SEC14L4	15.348	3.940	0.001	0.047
A_23_P63736	NR_026827	LOC84856	15.056	3.912	0.001	0.046
A_33_P3424577			15.036	3.910	0.000	0.046
A_24_P289383	NM_017780	CHD7	13.766	3.783	0.001	0.048
A_23_P82324	NM_032415	CARD11	13.653	3.771	0.000	0.040
A_32_P169406	NR_026656	LOC400043	13.556	3.761	0.000	0.021
A_33_P3263432	NM_003637	ITGA10	13.486	3.753	0.000	0.046
A_23_P40880	NM_178868	CMTM8	13.462	3.751	0.001	0.045
A_33_P3275973	NR_015365	LOC553137	13.423	3.747	0.000	0.033
A_23_P67453	NM_000363	TNNI3	13.401	3.744	0.000	0.048
A_23_P113034	NM_032024	C10orf11	13.237	3.726	0.000	0.000
A_23_P28466	NM_178821	WDR69	12.714	3.668	0.001	0.045
A_32_P87013	NM_000584	IL8	12.473	3.641	0.000	0.034
A_23_P216468	NM_004170	SLC1A1	12.385	3.631	0.001	0.045
A_33_P3260377	NM_031461	CRISPLD1	12.373	3.629	0.000	0.046
A_24_P88763	NM_032603	LOXL3	12.190	3.608	0.001	0.045
A_23_P157736	NM_032728	PPAPDC3	11.836	3.565	0.001	0.045
A_33_P3561747	NM_001145545	C16orf82	11.747	3.554	0.000	0.034
A_33_P3408757	NM_001291281.3	FOXO6	11.743	3.554	0.000	0.027
A_23_P129458	NM_145168	SDR42E1	11.668	3.544	0.000	0.021
A_23_P391396	NM_001005463	EBF3	11.466	3.519	0.001	0.047
A_33_P3275707	NM_001039569	AP1S3	11.435	3.515	0.001	0.046
A_24_P664850	NM_006011	ST8SIA2	11.354	3.505	0.000	0.033
A_23_P212042	NM_005929	MFI2	11.309	3.499	0.000	0.038
A_23_P369328	NM_145306	C10orf35	11.303	3.499	0.001	0.045
A_24_P270033	AK095399	MPZL3	11.055	3.467	0.001	0.045
A_23_P28120	NM_016932	SIX2	10.926	3.450	0.001	0.048
A_24_P166613	NM_017549	EPDR1	10.890	3.445	0.001	0.045
A_32_P99347	NR_024376	C9orf110	10.884	3.444	0.001	0.050
A_33_P3214343	NM_001134478	PLCXD2	10.639	3.411	0.000	0.039
A_24_P48057	NM_005853	IRX5	10.509	3.394	0.001	0.045
A_33_P3370875			10.492	3.391	0.000	0.024
A_33_P3358208	NM_013358	PADI1	10.386	3.377	0.001	0.050
A_32_P112623	XR_079078	LOC100293193	10.222	3.354	0.000	0.025
A_33_P3276693	NM_002632	PGF	10.141	3.342	0.000	0.020
A_33_P3273552	NM_002282	KRT83	10.094	3.335	0.001	0.045
A_33_P3258627			9.968	3.317	0.000	0.042
A_24_P158946	NM_139241	FGD4	9.940	3.313	0.001	0.045
A_23_P56559	NM_005771	DHRS9	9.855	3.301	0.000	0.024
A_32_P104063	XR_042100	CRNDE	9.776	3.289	0.000	0.042
A_23_P259207	NM_018271	THNSL2	9.745	3.285	0.000	0.046
A_33_P3263232	NM_030891	LRRC3	9.685	3.276	0.001	0.046
A_23_P11685	NM_024420	PLA2G4A	9.588	3.261	0.001	0.048
A_23_P32414	NM_016542	RP6-213H19.1	9.505	3.249	0.001	0.045
A_33_P3227793	NM_006569	CGREF1	9.397	3.232	0.000	0.036
A_23_P48109	NM_016533	NINJ2	9.307	3.218	0.001	0.048
A_32_P115050	XR_041422	LOC646576	9.277	3.214	0.000	0.023
A_23_P30243	NM_022350	ERAP2	9.255	3.210	0.001	0.045

A_23_P369994	NM_004734	DCLK1	9.001	3.170	0.001	0.046
A_33_P3409518	NR_027156	TUBBP5	8.936	3.160	0.001	0.045
A_32_P170481	NR_026658	LOC100240735	8.799	3.137	0.001	0.045
A_24_P56240	NM_153634	CPNE8	8.504	3.088	0.000	0.048
A_33_P3287158	NM_020742	NLGN4X	8.437	3.077	0.000	0.048
A_23_P421306	NM_177963	SYT12	8.414	3.073	0.001	0.049
A_23_P88880	NM_015069	ZNF423	8.383	3.067	0.001	0.045
A_23_P300150	NM_172387	NFATC1	8.346	3.061	0.001	0.045
A_23_P20532	AK024257	LOC642406	8.232	3.041	0.000	0.024
A_23_P145935	NM_004445	EPHB6	8.033	3.006	0.001	0.047
A_33_P3398196			7.912	2.984	0.000	0.023
A_23_P143935	NM_025163	PIGZ	7.699	2.945	0.001	0.046
A_23_P115726	NM_194298	SLC16A9	7.386	2.885	0.000	0.000
A_33_P3300965	NM_153693	HOXC6	7.267	2.861	0.001	0.046
A_23_P87709	NM_024829	PLBD1	7.210	2.850	0.001	0.046
A_23_P24129	NM_012242	DKK1	7.182	2.844	0.000	0.040
A_32_P211248	XM_001717925	LOC100131138	7.167	2.841	0.001	0.048
A_23_P162165	NM_023930	KCTD14	7.160	2.840	0.001	0.049
A_23_P202269	NM_020987	ANK3	7.090	2.826	0.001	0.047
A_23_P70968	NM_006896	HOXA7	7.020	2.812	0.001	0.047
A_23_P30913	NM_033554	HLA-DPA1	6.980	2.803	0.001	0.046
A_24_P49260	NM_018327	SPTLC3	6.920	2.791	0.001	0.047
A_24_P306443	NM_001033515	LOC100132288	6.891	2.785	0.000	0.046
A_23_P41390	NM_018986	SH3TC1	6.885	2.783	0.001	0.048
A_24_P150466	NM_001034852	SMOC1	6.878	2.782	0.000	0.028
A_33_P3227079	XM_005272231.2	PLPP7	6.846	2.775	0.001	0.048
A_23_P208788	NM_033520	C19orf33	6.843	2.775	0.001	0.048
A_33_P3209209	XM_001132904	LOC728694	6.782	2.762	0.001	0.045
A_23_P323143	NR_027788	ZNF767	6.775	2.760	0.001	0.045
A_33_P3297562	NM_033267	IRX2	6.666	2.737	0.001	0.047
A_23_P78018	NM_018672	ABCA5	6.423	2.683	0.000	0.025
A_33_P3381751	NM_003253	TIAM1	6.232	2.640	0.001	0.047
A_24_P371628	NM_054027	ANKH	6.223	2.638	0.001	0.048
A_32_P104746	NM_020972	ZFYVE28	6.156	2.622	0.001	0.048
A_23_P421175	NM_198488	FAM83H	6.127	2.615	0.001	0.048
A_24_P124558	NM_022658	HOXC8	5.869	2.553	0.001	0.048
A_32_P4018	AK000776	ROR1	5.813	2.539	0.000	0.043
A_23_P18372	NM_032047	B3GNT5	5.801	2.536	0.001	0.046
A_23_P211345	NM_080647	TBX1	5.780	2.531	0.001	0.048
A_32_P180265	XM_002342916	LOC100287241	5.774	2.529	0.001	0.048
A_23_P159775	NM_004961	GABRE	5.738	2.520	0.000	0.025
A_33_P3336617	NM_000382	ALDH3A2	5.730	2.519	0.000	0.033
A_23_P413641	NM_020820	PREX1	5.727	2.518	0.000	0.038
A_23_P146946	NM_001323	CST6	5.704	2.512	0.001	0.048
A_23_P75283	NM_006744	RBP4	5.677	2.505	0.001	0.048
A_23_P202206	NM_183239	GSTO2	5.567	2.477	0.001	0.048
A_33_P3336622	NM_001031806	ALDH3A2	5.534	2.468	0.001	0.048
A_33_P3311267	NM_181608	KRTAP19-2	5.532	2.468	0.001	0.048

A_24_P238131	NM_015551	SUSD5	5.518	2.464	0.001	0.048
A_23_P73429	NM_005335	HCLS1	5.385	2.429	0.000	0.025
A_24_P342096	NR_024060	FAM27A	5.363	2.423	0.001	0.048
A_32_P205110	NM_001453	FOXC1	5.240	2.390	0.001	0.048
A_24_P342829	NM_152527	SLC16A14	5.237	2.389	0.000	0.045
A_23_P259442	NM_001873	CPE	5.228	2.386	0.000	0.042
A_33_P3370521	XM_001723587	LOC100133008	5.193	2.377	0.001	0.048
A_23_P46470	NM_018948	ERRFI1	5.161	2.368	0.001	0.048
A_32_P86763	NM_004613	TGM2	5.110	2.353	0.000	0.034
A_33_P3377519	NM_024014	HOXA6	5.073	2.343	0.001	0.048
A_23_P309619	NM_001145206	KIAA1671	5.029	2.330	0.000	0.046
A_33_P3336257	NM_024337	IRX1	4.982	2.317	0.001	0.048
A_23_P126075	NM_002245	KCNK1	4.951	2.308	0.001	0.048
A_33_P3336686	NM_004669	CLIC3	4.742	2.245	0.001	0.049
A_23_P218646	NM_032945	TNFRSF6B	4.680	2.227	0.000	0.039
A_33_P3216438	NM_198546	SPATA21	4.668	2.223	0.000	0.029
A_23_P433111	NM_153706	C5orf35	4.515	2.175	0.001	0.050
A_32_P79483	XM_001128325	LOC283481	4.348	2.120	0.000	0.040
A_23_P32036	NM_017881	C9orf95	4.251	2.088	0.000	0.029
A_23_P5601	NM_001381	DOK1	4.245	2.086	0.000	0.048
A_33_P3310929	NM_003474	ADAM12	4.167	2.059	0.000	0.037
A_23_P120243	NM_024501	HOXD1	4.119	2.042	0.000	0.001
A_23_P58588	NM_003062	SLIT3	4.049	2.018	0.001	0.048
A_24_P109644	XR_017002	LOC220077	4.034	2.012	0.000	0.041
A_23_P161439	NM_006829	C10orf116	3.965	1.987	0.000	0.038
A_23_P69030	NM_001850	COL8A1	3.876	1.955	0.001	0.045
A_24_P412734	NM_173502	PRSS36	3.833	1.939	0.001	0.048
A_33_P3358601	NM_001170820.4	IFITM10	3.785	1.920	0.001	0.045
A_23_P315364	NM_002089	CXCL2	3.713	1.893	0.001	0.049
A_23_P72157	NM_032219	MFSD7	3.704	1.889	0.000	0.036
A_23_P113462	NM_017641	KIF21A	3.697	1.886	0.000	0.028
A_23_P502142	NM_002037	FYN	3.686	1.882	0.000	0.000
A_32_P208120	NM_153498	CAMK1D	3.680	1.880	0.001	0.048
A_23_P105524	NM_033123	PLCZ1	3.632	1.861	0.000	0.010
A_23_P210164	NM_019558	HOXD8	3.610	1.852	0.001	0.047
A_23_P96041	NM_032227	TMEM164	3.573	1.837	0.000	0.036
A_23_P153676	NM_003260	TLE2	3.489	1.803	0.000	0.046
A_23_P28434	NM_003761	VAMP8	3.366	1.751	0.000	0.046
A_24_P104119	NM_019034	RHOF	3.312	1.728	0.001	0.048
A_23_P151075	NM_001175	ARHGDIB	3.274	1.711	0.000	0.042
A_23_P67847	NM_024572	GALNT14	3.222	1.688	0.000	0.048
A_23_P359245	NM_000245	MET	3.143	1.652	0.000	0.000
A_23_P316612	NM_147193	GLIS1	3.142	1.652	0.001	0.049
A_23_P121527	NM_015990	KLHL5	3.138	1.650	0.000	0.000
A_23_P10194	NM_201575	SEZ6L2	3.114	1.639	0.000	0.047
A_24_P355649	NM_002017	FLI1	3.110	1.637	0.001	0.047
A_33_P3420446	NM_001161528	LOC401387	3.021	1.595	0.001	0.046
A_33_P3363305			3.020	1.594	0.001	0.045

A_23_P353574	NM_133494	NEK7	2.996	1.583	0.001	0.048
A_23_P101013	NM_007267	TMC6	2.977	1.574	0.000	0.000
A_23_P382188	NM_001013841	STAP2	2.955	1.563	0.000	0.046
A_32_P52785	NM_015345	DAAM2	2.953	1.562	0.000	0.047
A_23_P419202	NM_033160	ZNF658	2.948	1.560	0.000	0.046
A_32_P152437	NM_005100	AKAP12	2.931	1.551	0.001	0.045
A_33_P3399755			2.927	1.549	0.000	0.046
A_32_P150391	NM_001105520	C17orf100	2.915	1.543	0.001	0.048
A_23_P102890	NM_032476	MRPS6	2.910	1.541	0.000	0.025
A_24_P411749	NM_020455	GPR126	2.890	1.531	0.000	0.048
A_23_P85682	NM_005595	NFIA	2.843	1.507	0.001	0.048
A_23_P202117	BC007377	PCGF5	2.824	1.498	0.001	0.048
A_24_P861009	NM_001007246	BRWD1	2.809	1.490	0.001	0.046
A_33_P3296205	NM_183422	TSC22D1	2.803	1.487	0.000	0.047
A_33_P3341105	NM_002602	PDE6G	2.770	1.470	0.000	0.047
A_23_P51397	NM_001008493	ENAH	2.754	1.461	0.001	0.048
A_33_P3382887	XM_001714799	hCG_1659830	2.750	1.459	0.000	0.048
A_23_P120281	NM_022336	EDAR	2.745	1.457	0.000	0.042
A_33_P3629678	NM_000093	COL5A1	2.711	1.439	0.001	0.045
A_23_P143845	NM_015508	TIPARP	2.671	1.417	0.001	0.048
A_23_P76983	NM_025057	C14orf45	2.662	1.412	0.001	0.048
A_33_P3551349	AK127450	LOC439911	2.632	1.396	0.001	0.047
A_24_P134392	NM_006948	HSPA13	2.586	1.371	0.001	0.046
A_23_P101434	NM_033297	NLRP12	2.558	1.355	0.001	0.046
A_24_P122337	NM_080737	SYTL4	2.533	1.341	0.001	0.046
A_23_P254733	NM_024629	MLF1IP	2.495	1.319	0.001	0.048
A_33_P3411632	NM_025268	TMEM121	2.475	1.307	0.001	0.045
A_33_P3252359	NM_203314	BDH1	2.459	1.298	0.001	0.048
A_24_P235266	NM_001001555	GRB10	2.453	1.295	0.001	0.048
A_23_P40956	NM_016362	GHRL	2.453	1.295	0.000	0.044
A_23_P119778	NM_020342	SLC39A10	2.352	1.234	0.000	0.000
A_23_P401076	NM_145006	SUSD3	2.321	1.214	0.001	0.048
A_23_P381714	NM_198584	CA13	2.314	1.210	0.001	0.049
A_23_P38346	NM_024119	DHX58	2.313	1.210	0.001	0.048
A_23_P119040	NM_001142966	GREB1L	2.207	1.142	0.001	0.045
A_23_P120594	NM_032501	ACSS1	0.439	-1.189	0.000	0.036
A_33_P3263417	NR_015431	FLJ43663	0.421	-1.248	0.001	0.048
A_33_P3222367	NR_002988	SNORA74B	0.408	-1.293	0.001	0.046
A_24_P309095	NM_005045	RELN	0.395	-1.340	0.001	0.049
A_23_P121665	NM_020777	SORCS2	0.386	-1.373	0.000	0.000
A_33_P3212432	XM_002346043	LOC100293406	0.373	-1.422	0.001	0.047
A_23_P163455	NM_002373	MAP1A	0.368	-1.442	0.001	0.045
A_33_P3353791	NM_181501	ITGA1	0.364	-1.459	0.001	0.045
A_23_P8640	NM_001039966	GPER	0.356	-1.490	0.000	0.047
A_23_P255331	NM_032623	C4orf49	0.351	-1.511	0.000	0.047
A_33_P3844650	NM_012098	ANGPTL2	0.347	-1.527	0.001	0.048
A_23_P64792	NM_014505	KCNMB4	0.346	-1.531	0.000	0.046
A_32_P86739	NM_001010911	C10orf114	0.343	-1.543	0.000	0.046

A_32_P78816	NM_004577	PSPH	0.342	-1.546	0.000	0.046
A_32_P167239	NM_152406	AFAP1L1	0.329	-1.603	0.001	0.048
A_33_P3413701	NM_001040458	ERAP1	0.328	-1.607	0.000	0.046
A_23_P70670	NM_004233	CD83	0.324	-1.626	0.000	0.048
A_23_P151805	NM_006329	FBLN5	0.321	-1.640	0.000	0.043
A_23_P47034	NM_002729	HHEX	0.315	-1.668	0.000	0.046
A_33_P3223495			0.305	-1.712	0.001	0.048
A_23_P74887	NM_014654	SDC3	0.305	-1.715	0.000	0.044
A_33_P3255209	NR_024275	LOC151162	0.299	-1.742	0.000	0.048
A_33_P3334443	NM_001006605	FAM69A	0.295	-1.762	0.000	0.042
A_24_P6903	NM_001017992	ACTBL2	0.295	-1.763	0.000	0.044
A_24_P11315	NM_020190	OLFML3	0.294	-1.766	0.000	0.046
A_33_P3262191	NM_014427	CPNE7	0.291	-1.782	0.000	0.044
A_33_P3334448	NR_002324	SNORA62	0.290	-1.784	0.000	0.046
A_23_P101374	NM_030622	CYP2S1	0.283	-1.820	0.000	0.036
A_33_P3337485	NM_020404	CD248	0.275	-1.864	0.000	0.038
A_23_P42306	NM_006120	HLA-DMA	0.273	-1.871	0.001	0.049
A_23_P66017	NM_145239	PRRT2	0.273	-1.874	0.001	0.045
A_33_P3376095	NM_001040709	SYPL2	0.272	-1.878	0.000	0.046
A_24_P8220	NM_004807	HS6ST1	0.267	-1.903	0.000	0.039
A_33_P3332081	NM_006558	KHDRBS3	0.260	-1.942	0.000	0.040
A_33_P3344282			0.260	-1.942	0.000	0.038
A_33_P3278362	NM_020349	ANKRD2	0.260	-1.946	0.000	0.037
A_32_P189790	NR_027134	LINC02716	0.258	-1.956	0.000	0.046
A_23_P105562	NM_000552	VWF	0.256	-1.969	0.001	0.049
A_33_P3417195	NM_203425	C17orf82	0.255	-1.973	0.001	0.045
A_24_P215653	NM_175060	CLEC14A	0.252	-1.987	0.000	0.046
A_23_P16743	NM_002410	MGAT5	0.252	-1.988	0.000	0.048
A_33_P3257861	NM_001134707	SARDH	0.248	-2.010	0.001	0.048
A_24_P254949	NM_021965	PGM5	0.230	-2.119	0.000	0.032
A_23_P43276	NM_032777	GPR124	0.220	-2.185	0.000	0.047
A_33_P3245126	NM_182757.4	RNF144B	0.219	-2.193	0.001	0.045
A_33_P3393821	NM_001733	C1R	0.218	-2.195	0.000	0.036
A_24_P896205	XM_944447	LOC645722	0.213	-2.234	0.001	0.050
A_33_P3323718	NM_001008224	UACA	0.207	-2.272	0.001	0.050
A_23_P71328	NM_030583	MATN2	0.195	-2.356	0.001	0.049
A_23_P257043	NM_005261	GEM	0.195	-2.361	0.000	0.042
A_23_P34233	NM_014298	QPRT	0.195	-2.361	0.001	0.047
A_33_P3271276	NM_001130014	PSG5	0.193	-2.374	0.000	0.046
A_23_P145606	NM_001006630	CHRM2	0.191	-2.386	0.000	0.048
A_23_P350005	NM_173553	TRIML2	0.191	-2.390	0.001	0.048
A_33_P3275846	NM_001127222	CACNA1A	0.190	-2.395	0.000	0.027
A_23_P394395	NM_020433	JPH2	0.187	-2.417	0.000	0.037
A_23_P127911	NM_015430	PAMR1	0.186	-2.424	0.001	0.048
A_23_P50946	NM_005855	RAMP1	0.186	-2.426	0.001	0.048
A_32_P112493	NM_138370	SGK493	0.184	-2.443	0.001	0.048
A_23_P143817	NM_053025	MYLK	0.183	-2.448	0.001	0.048
A_23_P205177	NM_000504	F10	0.181	-2.462	0.001	0.049

A_23_P500614	NM_001243	TNFRSF8	0.180	-2.473	0.000	0.039
A_24_P75190	NM_000519	HBD	0.180	-2.474	0.000	0.025
A_23_P3552	BC009198	LOC730092	0.180	-2.474	0.001	0.048
A_23_P82929	NM_002514	NOV	0.180	-2.474	0.001	0.048
A_23_P134854	NM_194284	CLDN23	0.179	-2.485	0.001	0.048
A_23_P200710	NM_002646	PIK3C2B	0.175	-2.512	0.001	0.048
A_33_P3395605	NM_181724	TMEM119	0.175	-2.514	0.001	0.048
A_23_P205713	NM_014178	STXBP6	0.174	-2.526	0.001	0.048
A_23_P217917	NM_147148	GSTM4	0.170	-2.558	0.001	0.048
A_33_P3276718	NM_001010934	HGF	0.165	-2.596	0.001	0.048
A_24_P3005	NM_002977	SCN9A	0.162	-2.623	0.001	0.048
A_23_P400515	NM_020816	KIF17	0.162	-2.628	0.000	0.024
A_33_P3402329	NR_024607	MGC16121	0.161	-2.637	0.001	0.048
A_24_P319736	NM_002398	MEIS1	0.161	-2.638	0.001	0.048
A_23_P75800	NM_013401	RAB3IL1	0.158	-2.663	0.001	0.048
A_23_P256205	NM_014945	ABLIM3	0.150	-2.737	0.001	0.047
A_23_P78795	NM_001009813	MEIS3	0.149	-2.744	0.001	0.049
A_23_P307392	AK024141	DPF3	0.144	-2.799	0.000	0.037
A_33_P3413468	NM_021783	EDA2R	0.144	-2.801	0.001	0.048
A_23_P349966	NM_152913	TMEM130	0.143	-2.804	0.000	0.031
A_23_P399255	NM_152737	RNF182	0.143	-2.809	0.000	0.038
A_33_P3276713	NM_001010931	HGF	0.141	-2.823	0.001	0.047
A_23_P432573	NM_145015	MRGPRF	0.140	-2.837	0.000	0.023
A_23_P29953	NM_172174	IL15	0.139	-2.850	0.000	0.024
A_33_P3363560	NM_001136216	TMEM51	0.139	-2.851	0.000	0.047
A_23_P53193	NM_206927	SYTL2	0.137	-2.867	0.000	0.024
A_23_P216307	NM_004349	RUNX1T1	0.137	-2.870	0.001	0.047
A_24_P709377	NR_015377	LOC654433	0.135	-2.885	0.001	0.047
A_23_P112482	NM_004925	AQP3	0.135	-2.889	0.000	0.000
A_23_P110430	NM_002448	MSX1	0.131	-2.929	0.000	0.000
A_32_P74409	NM_001145033	LOC387763	0.128	-2.960	0.000	0.045
A_33_P3418541			0.128	-2.964	0.000	0.046
A_33_P3315375	NM_001104587	SLFN11	0.124	-3.011	0.001	0.046
A_32_P160561	NM_152721	DOK6	0.124	-3.015	0.001	0.047
A_33_P3342967	XR_078986	LOC100129186	0.123	-3.028	0.001	0.047
A_32_P57810	NM_052916	RNF157	0.120	-3.060	0.000	0.040
A_33_P3234020	NM_004884	IGDCC3	0.119	-3.075	0.000	0.040
A_23_P258136	NM_015419	MXRA5	0.118	-3.088	0.000	0.022
A_23_P361085	NR_003038	SNHG5	0.116	-3.113	0.001	0.045
A_23_P301855	NM_002338	LSAMP	0.115	-3.118	0.001	0.048
A_24_P328524	NM_003947	KALRN	0.113	-3.143	0.000	0.044
A_23_P408376	NM_025015	HSPA12A	0.110	-3.178	0.000	0.024
A_23_P69326	NM_183393	CADPS	0.109	-3.192	0.000	0.038
A_24_P128442	NM_152380	TBX15	0.109	-3.201	0.000	0.046
A_23_P373521	NM_021973	HAND2	0.109	-3.202	0.001	0.049
A_23_P145054	NM_001085480	FAM162B	0.107	-3.218	0.001	0.045
A_33_P3378514	NM_001083	PDE5A	0.103	-3.284	0.000	0.025
A_23_P91943	NM_000882	IL12A	0.100	-3.315	0.000	0.047

A_24_P71649	NM_020872	CNTN3	0.099	-3.340	0.001	0.045
A_33_P3825869	NM_199460	CACNA1C	0.098	-3.345	0.000	0.024
A_23_P49816	NM_018404	ADAP2	0.097	-3.365	0.001	0.045
A_23_P40295	NM_012261	C20orf103	0.096	-3.380	0.000	0.036
A_33_P3424062	NM_002236	KCNF1	0.093	-3.428	0.001	0.045
A_23_P164451	NM_005994	TBX2	0.091	-3.465	0.000	0.019
A_33_P3238415	NM_001080554	GSG1	0.090	-3.479	0.001	0.045
A_23_P216966	NM_000962	PTGS1	0.087	-3.516	0.000	0.019
A_24_P892472	NR_002791	EMX2OS	0.077	-3.701	0.000	0.040
A_33_P3351298			0.077	-3.702	0.001	0.045
A_23_P252082	NM_018487	TMEM176A	0.074	-3.748	0.001	0.045
A_33_P3302125	NM_178428	LCE2A	0.074	-3.751	0.001	0.045
A_33_P3325723	NM_001822	CHN1	0.074	-3.755	0.000	0.039
A_23_P139864	NM_031289	GSG1	0.073	-3.769	0.001	0.045
A_23_P159237	NM_005293	GPR20	0.073	-3.773	0.000	0.020
A_33_P3850216	NM_033058	TRIM55	0.073	-3.775	0.001	0.048
A_23_P328074	NM_002968	SALL1	0.073	-3.776	0.001	0.047
A_33_P3343442	XR_078693	ZNF316	0.071	-3.819	0.001	0.045
A_32_P101917		LOC283904	0.069	-3.849	0.001	0.045
A_23_P51019	NM_021007	SCN2A	0.069	-3.856	0.001	0.048
A_23_P208866	NM_004877	GMFG	0.065	-3.933	0.000	0.021
A_33_P3587376	NR_024214	SNAR-A3	0.065	-3.950	0.001	0.046
A_33_P3287223	NM_001935	DPP4	0.062	-4.003	0.000	0.046
A_33_P3329043	XM_001724293	LOC100133889	0.062	-4.005	0.000	0.033
A_33_P3315134	NR_026597	DIRC3	0.061	-4.036	0.001	0.049
A_23_P74609	NM_015714	G0S2	0.059	-4.088	0.000	0.046
A_33_P3347291	NM_006774	INMT	0.058	-4.100	0.000	0.046
A_23_P215744	NM_033427	CTTNBP2	0.058	-4.107	0.001	0.046
A_23_P97402	NM_020439	CAMK1G	0.058	-4.119	0.000	0.028
A_23_P91283	NM_020356	CASS4	0.057	-4.131	0.000	0.027
A_33_P3387646	BC052945	LOC643201	0.055	-4.190	0.000	0.048
A_23_P56703	NM_001080824	C2orf89	0.050	-4.313	0.000	0.041
A_23_P500353	NM_021614	KCNN2	0.048	-4.370	0.000	0.046
A_23_P39955	NM_001615	ACTG2	0.048	-4.384	0.000	0.028
A_33_P3314176	NM_017709	FAM46C	0.046	-4.428	0.000	0.046
A_23_P110957	NM_001452	FOXF2	0.046	-4.450	0.000	0.000
A_23_P374844	NM_015973	GAL	0.043	-4.554	0.001	0.047
A_24_P192805	NM_001007232	CARD17	0.040	-4.651	0.000	0.046
A_23_P304897	NM_000623	BDKRB2	0.040	-4.652	0.000	0.045
A_33_P3290532	AK091766	LOC255480	0.040	-4.657	0.001	0.045
A_33_P3316786	NM_080759	DACH1	0.039	-4.669	0.001	0.045
A_23_P64173	NM_001017534	CARD16	0.037	-4.738	0.000	0.024
A_23_P44264	NM_004098	EMX2	0.037	-4.751	0.001	0.045
A_23_P63521	NM_178429	LCE2C	0.036	-4.790	0.001	0.047
A_32_P169179	NR_002307	MSX2P1	0.036	-4.798	0.001	0.046
A_23_P400449	NM_020927	VAT1L	0.035	-4.826	0.000	0.018
A_23_P121533	NM_012445	SPON2	0.032	-4.960	0.000	0.000
A_33_P3240328	NM_002653	PITX1	0.029	-5.127	0.000	0.046

A_23_P157007	NM_014020	TMEM176B	0.029	-5.132	0.000	0.046
A_23_P133386	NM_006909	RASGRF2	0.028	-5.179	0.001	0.045
A_23_P128744	NM_000710	BDKRB1	0.026	-5.269	0.000	0.046
A_33_P3357002	NM_178540	C1QTNF9	0.025	-5.313	0.001	0.046
A_33_P3396831			0.025	-5.322	0.000	0.019
A_23_P31755	NM_000756	CRH	0.023	-5.437	0.001	0.046
A_23_P431268	NM_014935	PLEKHA6	0.021	-5.540	0.000	0.046
A_33_P3238290	NM_080829	FAM65C	0.021	-5.547	0.000	0.046
A_23_P103601	NM_020379	MAN1C1	0.021	-5.603	0.000	0.046
A_23_P72770	NM_032147	USP44	0.019	-5.743	0.000	0.046
A_24_P102293	NM_015567	SLITRK5	0.018	-5.776	0.000	0.046
A_23_P126836	NM_003326	TNFSF4	0.017	-5.849	0.000	0.047
A_23_P134237	NM_002889	RARRES2	0.015	-6.093	0.000	0.010
A_24_P30557	NM_000192	TBX5	0.010	-6.608	0.000	0.046
A_23_P409093	NM_178826	ANO4	0.010	-6.644	0.000	0.015
A_23_P118254	NM_001451	FOXF1	0.010	-6.644	0.000	0.000

Supplementary Table S3. List of enriched biological processes with genes up-regulated more than two folds in hBMSC against hEF and FDR<0.05.

GO Term #	Term Description	Strength	FDR	Matching Proteins
Cluster 1				
GO:0060325	*Face morphogenesis*	*1.39*	*0.0338*	*DLX5,CRISPLD1,TBX1,DKK1*
GO:0009954	*Proximal/distal pattern formation*	*1.36*	*0.0399*	*IRX1,HOXB9,IRX3,HOXA9*
GO:0060324	*Face development*	*1.30*	*0.0092*	*DLX5,CRISPLD1,TBX1,DKK1,CHD7*
GO:0048704	*Embryonic skeletal system morphogenesis*	*1.25*	*0.0000*	*HOXA6,MDFI,HOXA7,SIX2,HOXB9,TBX1,HOXA9,IRX5,HOXC4*
GO:0048706	*Embryonic skeletal system development*	*1.21*	*0.0000*	*HOXA6,MDFI,HOXA7,HOXC6,SIX2,HOXB9,TBX1,HOXA9,NKX3-2,IRX5,HOXC4*
GO:0060415	Muscle tissue morphogenesis	1.10	0.0497	TBX1,TNNI3,ANKRD1,FOXC1,CHD7
GO:0009952	Anterior/posterior pattern specification	1.03	0.0000	HOXC8,HOXA6,HOXA7,HOXC6,MEOX2,SIX2,HOXB9,TBX1,HOXA9,DKK1,FOXC1,HOXC4
GO:0048705	Skeletal system morphogenesis	1.01	0.0000	HOXC8,DLX5,HOXA6,MDFI,HOXA7,SIX2,HOXB9,TBX1,HOXA9,FOXC1,NKX3-2,IRX5,HOXC4
Cluster 2				
GO:0032715	*Negative regulation of interleukin-6 production*	*1.29*	*0.0082*	*FOXJ1,GHRL,TLR4,NLRP12,CD200*
GO:0030593	*Neutrophil chemotaxis*	*1.06*	*0.0356*	*CCL11,CXCL8,PREX1,ITGB2,CXCL2*
GO:0050709	*Negative regulation of protein secretion*	*1.02*	*0.0475*	*SRGN,GHRL,SYTL4,NLRP12,CD200*

* The biological processes were identified by String analysis at https://string-db.org/. Two-cluster K-means clustering was applied here. Results shown are biological processes with enrichment strength >1 (log(enrichment)).

Supplementary Table S4. List of enriched biological processes with genes down-regulated more than two folds in hBMSC against hEF and FDR<0.05.

GO Term #	Term Description	Strength	FDR	Matching Proteins
Cluster 1				
KEGG_ hsa04610	*Complement and coagulation cascades*	*1.19*	*0.0106*	*BDKRB1,VWF,F10,C1R,BDKRB2*
Cluster 2				
GO:0003197	*Endocardial cushion development*	*1.41*	*0.0397*	*TBX2,FOXF1,TBX5,MSX1*
GO:0030326	*Embryonic limb morphogenesis*	*1.19*	*0.0078*	*TBX2,SALL1,PITX1,CACNA1C,TBX 5,HAND2,MSX1*
GO:0001508	*Action potential*	*1.16*	*0.0432*	*KCNMB4,CACNA1C,SCN2A,SCN9A ,KCNN2*

* The biological processes were identified by String analysis at https://string-db.org/. Two-cluster K-means clustering was applied here. Results shown are biological processes with enrichment strength >1 (log(enrichment)).

Supplementary Table S5. List of enriched biological processes with transcription factors differentially expressed more than 2 folds between hBMSC and hEF and FDR<0.05.

GO Term #	Term Description	Strength	FDR	Matching Proteins
Cluster 1				
GO:0010944	Negative regulation of transcription by competitive promoter binding	2.19	0.0226	HHEX,DACH1
GO:0060044	Negative regulation of cardiac muscle cell proliferation	2.05	0.0384	MEIS1,TBX5
GO:0035855	Megakaryocyte development	2.00	0.0470	MEIS1,FLI1
GO:0060216	Definitive hemopoiesis	2.00	0.0470	MEIS1,HOXA9
GO:0048706	*Embryonic skeletal system development*	*1.72*	*0.0000*	*TBX15,HOXA6,HOXA7,HOXC6,HOXB9,HOXD1,HOXA9,HOXC4*
GO:0048704	*Embryonic skeletal system morphogenesis*	*1.72*	*0.0000*	*TBX15,HOXA6,HOXA7,HOXB9,HOXA9,HOXC4*
GO:0009952	*Anterior/posterior pattern specification*	*1.60*	*0.0000*	*HOXC8,HOXA6,HOXA7,HOXC6,HHEX,HOXB9,HOXD8,HOXA9,HOXC4,EMX2*
GO:0003002	Regionalization	1.45	0.0000	HOXC8,HOXA6,HOXA7,HOXC6,HHEX,HOXB9,HOXD8,FOXJ1,HOXA9,HOXC4,EMX2
GO:0048705	Skeletal system morphogenesis	1.45	0.0000	HOXC8,TBX15,HOXA6,HOXA7,HOXB9,HOXD8,HOXA9,HOXC4
GO:1903707	Negative regulation of hemopoiesis	1.44	0.0003	HOXA7,MEIS1,FOXJ1,HOXA9,RUNX3
GO:0045638	Negative regulation of myeloid cell differentiation	1.42	0.0453	HOXA7,MEIS1,HOXA9
GO:0007389	Pattern specification process	1.41	0.0000	HOXC8,HOXA6,HOXA7,HOXC6,MEIS1,HHEX,HOXB9,TBX5,HOXD8,FOXJ1,HOXA9,HOXC4,EMX2
GO:0001501	Skeletal system development	1.35	0.0000	HOXC8,TBX15,HOXA6,HOXA7,HOXC6,MEIS1,HOXB9,HOXD8,HOXD1,HOXA9,RUNX3,HOXC4,FLI1
GO:0048562	Embryonic organ morphogenesis	1.30	0.0000	TBX15,HOXA6,HOXA7,FOXF2,HOXB9,HOXA9,HOXC4
GO:0009887	Animal organ morphogenesis	1.12	0.0000	HOXC8,TBX15,HOXA6,HOXA7,FOXF2,MEIS1,HOXB9,TBX5,HOXD8,FOXJ1,HOXA9,HOXC4,FLI1,EMX2,TLE2
GO:0048598	Embryonic morphogenesis	1.08	0.0001	TBX15,HOXA6,HOXA7,FOXF2,HOXB9,TBX5,HOXA9,HOXC4
GO:0000122	Negative regulation of	1.02	0.0000	HOXC8,TBX15,HOXA7,H

	transcription by rna polymerase ii			HEX,GLIS1,TBX5,HOXD8,FOXJ1,RUNX3,EMX2,DACH1
GO:0009790	Embryo development	1.01	0.0000	TBX15,HOXA6,HOXA7,HOXC6,FOXF2,MEIS1,HOXB9,TBX5,HOXD8,HOXD1,HOXA9,HOXC4
Cluster 2				
GO:0072086	Specification of loop of henle identity	2.68	0.0022	IRX1,IRX3
GO:0003337	Mesenchymal to epithelial transition involved in metanephros morphogenesis	2.26	0.0082	SALL1,SIX2
GO:0042473	Outer ear morphogenesis	2.21	0.0098	SALL1,TBX1
GO:0060982	Coronary artery morphogenesis	2.21	0.0098	TBX1,HAND2
GO:0010463	Mesenchymal cell proliferation	2.13	0.0003	SIX2,HAND2,MSX1
GO:0007379	Segment specification	2.11	0.0004	MEOX2,IRX1,IRX3
GO:0021889	Olfactory bulb interneuron differentiation	2.08	0.0146	DLX5,SALL1
GO:2001053	Regulation of mesenchymal cell apoptotic process	2.08	0.0146	TBX1,MSX1
GO:0042474	Middle ear morphogenesis	2.04	0.0006	SIX2,TBX1,MSX1
GO:0042693	Muscle cell fate commitment	1.99	0.0200	TBX2,TBX1
GO:0043517	Positive regulation of dna damage response, signal transduction by p53 class mediator	1.96	0.0222	ANKRD1,MSX1
GO:0072189	Ureter development	1.93	0.0244	FOXF1,NFIA
GO:0048557	Embryonic digestive tract morphogenesis	1.91	0.0269	FOXF1,SIX2
GO:0060325	Face morphogenesis	1.85	0.0017	DLX5,TBX1,MSX1
GO:0072210	Metanephric nephron development	1.85	0.0017	SALL1,SIX2,IRX1
GO:0010464	Regulation of mesenchymal cell proliferation	1.82	0.0021	FOXF1,TBX1,NFIB
GO:0001945	Lymph vessel development	1.82	0.0375	TBX1,FOXC1
GO:0048566	Embryonic digestive tract development	1.81	0.0022	SALL1,FOXF1,SIX2
GO:0110111	Negative regulation of animal organ morphogenesis	1.81	0.0022	TBX2,FOXC1,NFIB
GO:0043586	Tongue development	1.80	0.0403	TBX1,HAND2
GO:0048844	Artery morphogenesis	1.77	0.0000	TBX2,FOXF1,TBX1,HAND2,FOXC1
GO:0072132	Mesenchyme morphogenesis	1.77	0.0001	TBX2,FOXF1,FOXC1,MSX1
GO:0002053	Positive regulation of mesenchymal cell proliferation	1.76	0.0464	FOXF1,TBX1
GO:0060021	Roof of mouth development	1.75	0.0000	DLX5,TBX2,MEOX2,TBX1,HAND2,MSX1,DLX6

GO:0048333	Mesodermal cell differentiation	1.75	0.0495	FOXF1,SIX2
GO:0043392	Negative regulation of dna binding	1.71	0.0002	MDFI,HAND2,NFIB,MSX1
GO:0021983	Pituitary gland development	1.71	0.0036	SALL1,PITX1,MSX1
GO:2000826	Regulation of heart morphogenesis	1.71	0.0036	TBX2,HAND2,FOXC1
GO:0003197	Endocardial cushion development	1.69	0.0040	TBX2,FOXF1,MSX1
GO:0042733	Embryonic digit morphogenesis	1.68	0.0003	TBX2,SALL1,HAND2,MSX1
GO:0048701	Embryonic cranial skeleton morphogenesis	1.66	0.0050	SIX2,TBX1,IRX5
GO:0072088	Nephron epithelium morphogenesis	1.64	0.0004	SALL1,SIX2,IRX1,IRX3
GO:0030326	Embryonic limb morphogenesis	1.60	0.0000	DLX5,TBX2,SALL1,PITX1,HAND2,MSX1,DLX6
GO:2000677	Regulation of transcription regulatory region dna binding	1.59	0.0073	HAND2,FOXC1,MSX1
GO:0035282	Segmentation	1.57	0.0000	MEOX2,FOXF1,IRX1,IRX3,FOXC1
GO:0072009	Nephron epithelium development	1.57	0.0000	SALL1,SIX2,IRX1,IRX3,FOXC1
GO:0035050	Embryonic heart tube development	1.56	0.0007	TBX2,TBX1,HAND2,FOXC1
GO:0042471	Ear morphogenesis	1.55	0.0000	DLX5,SALL1,SIX2,TBX1,MSX1,DLX6
GO:0042475	Odontogenesis of dentin-containing tooth	1.55	0.0007	TBX1,HAND2,FOXC1,MSX1
GO:0001656	Metanephros development	1.54	0.0008	SALL1,SIX2,IRX1,IRX3
GO:0001947	Heart looping	1.53	0.0102	TBX2,TBX1,HAND2
GO:0072078	Nephron tubule morphogenesis	1.53	0.0102	SALL1,IRX1,IRX3
GO:0001756	Somitogenesis	1.52	0.0110	MEOX2,FOXF1,FOXC1
GO:0060173	Limb development	1.51	0.0000	DLX5,TBX2,SALL1,MEOX2,PITX1,HAND2,MSX1,DLX6
GO:0001707	Mesoderm formation	1.51	0.0114	FOXF1,SIX2,FOXC1
GO:0007498	Mesoderm development	1.49	0.0001	FOXF1,SIX2,IRX3,TBX1,FOXC1
GO:0048704	Embryonic skeletal system morphogenesis	1.48	0.0014	MDFI,SIX2,TBX1,IRX5
GO:0003208	Cardiac ventricle morphogenesis	1.48	0.0134	FOXF1,HAND2,FOXC1
GO:0003151	Outflow tract morphogenesis	1.47	0.0138	TBX2,TBX1,HAND2
GO:0014032	Neural crest cell development	1.46	0.0150	TBX1,HAND2,FOXC1
GO:0060415	Muscle tissue morphogenesis	1.46	0.0150	TBX1,ANKRD1,FOXC1
GO:0003206	Cardiac chamber morphogenesis	1.45	0.0002	TBX2,FOXF1,TBX1,HAND2,FOXC1
GO:0051101	Regulation of dna binding	1.45	0.0002	MDFI,HAND2,FOXC1,NFIB,MSX1

GO:0048562	Embryonic organ morphogenesis	1.42	0.0000	DLX5,MDFI,TBX2,SALL1,FOXF1,SIX2,TBX1,HAND2,MSX1,IRX5,DLX6
GO:0003205	Cardiac chamber development	1.41	0.0000	TBX2,SALL1,FOXF1,TBX1,HAND2,FOXC1
GO:0001708	Cell fate specification	1.40	0.0205	TBX2,SIX2,TBX1
GO:0060485	*Mesenchyme development*	*1.38*	*0.0000*	*TBX2,FOXF1,SIX2,TBX1,HAND2,FOXC1,MSX1*
GO:0048762	*Mesenchymal cell differentiation*	*1.38*	*0.0003*	*SIX2,TBX1,HAND2,FOXC1,MSX1*
GO:0007368	Determination of left/right symmetry	1.38	0.0029	TBX2,FOXF1,TBX1,HAND2
GO:0035270	Endocrine system development	1.38	0.0029	SALL1,PITX1,TBX1,MSX1
GO:0048863	*Stem cell differentiation*	*1.37*	*0.0004*	*TBX2,TBX1,HAND2,FOXC1,MSX1*
GO:0048565	Digestive tract development	1.35	0.0035	TBX2,SALL1,FOXF1,SIX2
GO:0003231	Cardiac ventricle development	1.35	0.0036	SALL1,FOXF1,HAND2,FOXC1
GO:0090596	Sensory organ morphogenesis	1.34	0.0000	DLX5,TBX2,SALL1,SIX2,TBX1,MSX1,IRX5,DLX6
GO:0048705	*Skeletal system morphogenesis*	*1.33*	*0.0000*	*DLX5,MDFI,SIX2,TBX1,FOXC1,MSX1,IRX5*
GO:0051216	*Cartilage development*	*1.33*	*0.0005*	*PITX1,SIX2,HAND2,NFIB,MSX1*
GO:0042472	Inner ear morphogenesis	1.32	0.0315	DLX5,TBX1,DLX6
GO:0003007	Heart morphogenesis	1.31	0.0000	TBX2,FOXF1,TBX1,HAND2,ANKRD1,FOXC1,MSX1
GO:0009952	Anterior/posterior pattern specification	1.31	0.0001	MEOX2,FOXF1,SIX2,TBX1,FOXC1,MSX1
GO:0007389	Pattern specification process	1.30	0.0000	MDFI,TBX2,MEOX2,FOXF1,SIX2,IRX1,IRX3,TBX1,HAND2,FOXC1,MSX1,MEIS3
GO:2000027	Regulation of animal organ morphogenesis	1.30	0.0000	TBX2,SIX2,TBX1,HAND2,FOXC1,NFIB,MSX1
GO:0060349	Bone morphogenesis	1.30	0.0366	DLX5,FOXC1,MSX1
GO:0048568	Embryonic organ development	1.29	0.0000	DLX5,MDFI,TBX2,SALL1,FOXF1,SIX2,TBX1,HAND2,FOXC1,MSX1,IRX5,DLX6
GO:0003002	Regionalization	1.29	0.0000	MDFI,MEOX2,FOXF1,SIX2,IRX1,IRX3,TBX1,FOXC1,MSX1
GO:0003279	Cardiac septum development	1.29	0.0382	TBX2,SALL1,TBX1
GO:0048598	Embryonic morphogenesis	1.28	0.0000	DLX5,MDFI,TBX2,SALL1,FOXF1,PITX1,SIX2,IRX1,IRX3,TBX1,HAND2,FOXC1,MSX1,IRX5,DLX6
GO:0072001	Renal system development	1.26	0.0000	SALL1,FOXF1,SIX2,IRX1

				,IRX3,NFIA,FOXC1
GO:0007219	Notch signaling pathway	1.26	0.0465	TBX2,FOXC1,ZNF423
GO:0060562	Epithelial tube morphogenesis	1.22	0.0000	TBX2,SALL1,FOXF1,IRX1,IRX3,TBX1,HAND2
GO:0001501	Skeletal system development	1.20	0.0000	DLX5,MDFI,PITX1,SIX2,TBX1,HAND2,FOXC1,NFIB,MSX1,IRX5,DLX6
GO:0048732	Gland development	1.20	0.0000	TBX2,SALL1,FOXF1,PITX1,TBX1,HAND2,FOXC1,NFIB,MSX1
GO:0007517	Muscle organ development	1.17	0.0004	MEOX2,PITX1,TBX1,ANKRD1,FOXC1,MSX1
GO:0048729	Tissue morphogenesis	1.15	0.0000	TBX2,SALL1,FOXF1,SIX2,IRX1,IRX3,TBX1,HAND2,ANKRD1,FOXC1,MSX1
GO:0007423	Sensory organ development	1.15	0.0000	DLX5,TBX2,SALL1,SIX2,TBX1,HAND2,FOXC1,MSX1,IRX5,DLX6,MEIS3
GO:0002009	Morphogenesis of an epithelium	1.12	0.0000	TBX2,SALL1,FOXF1,SIX2,IRX1,IRX3,TBX1,HAND2
GO:0009887	Animal organ morphogenesis	1.11	0.0000	DLX5,MDFI,TBX2,SALL1,FOXF1,SIX2,IRX1,IRX3,TBX1,HAND2,ANKRD1,FOXC1,NFIB,MSX1,IRX5,DLX6,MEIS3
GO:0014706	Striated muscle tissue development	1.11	0.0042	TBX2,MEOX2,PITX1,ANKRD1,FOXC1
GO:0007507	Heart development	1.10	0.0000	TBX2,SALL1,FOXF1,TBX1,HAND2,ANKRD1,FOXC1,MSX1,NFATC1
GO:0009790	Embryo development	1.09	0.0000	DLX5,MDFI,TBX2,SALL1,MEOX2,FOXF1,PITX1,SIX2,IRX1,IRX3,TBX1,HAND2,FOXC1,MSX1,IRX5,DLX6,MEIS3
GO:0048646	Anatomical structure formation involved in morphogenesis	1.09	0.0000	DLX5,TBX2,SALL1,MEOX2,FOXF1,SIX2,IRX1,IRX3,TBX1,HAND2,ANKRD1,FOXC1,NFIB,MSX1,DLX6
GO:0035239	Tube morphogenesis	1.08	0.0000	TBX2,SALL1,MEOX2,FOXF1,SIX2,IRX1,IRX3,TBX1,HAND2,FOXC1,NFIB
GO:0061061	Muscle structure development	1.08	0.0000	TBX2,MEOX2,FOXF1,PITX1,TBX1,ANKRD1,FOXC1,MSX1
GO:0042692	Muscle cell differentiation	1.07	0.0272	TBX2,FOXF1,TBX1,ANKRD1
GO:0045165	Cell fate commitment	1.07	0.0288	TBX2,PITX1,SIX2,TBX1
GO:0000122	Negative regulation of transcription by rna polymerase ii	1.05	0.0000	MDFI,TBX2,SALL1,FOXF1,IRX1,IRX3,TBX1,NFIA,ANKRD1,FOXC1,NFIB,IR

				X2,MSX1,IRX5
GO:0048514	Blood vessel morphogenesis	1.03	0.0022	TBX2,MEOX2,FOXF1,TBX1,HAND2,FOXC1
GO:0045892	Negative regulation of transcription, dna-templated	1.01	0.0000	MDFI,TBX2,SALL1,FOXF1,PITX1,SIX2,IRX1,IRX3,TBX1,NFIA,ANKRD1,FOXC1,NFIB,IRX2,MSX1,IRX5,ZNF423,ZNF658
GO:0035295	Tube development	1.01	0.0000	TBX2,SALL1,MEOX2,FOXF1,SIX2,IRX1,IRX3,TBX1,HAND2,NFIA,FOXC1,NFIB
GO:0043009	Chordate embryonic development	1.01	0.0000	MDFI,MEOX2,FOXF1,SIX2,TBX1,HAND2,FOXC1,MSX1,IRX5

* The biological processes were identified by String analysis at https://string-db.org/. Two-cluster K-means clustering was applied here. Results shown are biological processes with enrichment strength >1 (log(enrichment)).
